# Alarming decline in the carbon sink of European forests driven by disturbances

**DOI:** 10.1093/nsr/nwag354

**Published:** 2026-06-11

**Authors:** François Ritter, Philippe Ciais, Cornelius Senf, Maurizio Santoro, Yidi Xu, Agnès Pelissier-Tanon, Martin Schwartz, Ibrahim Fayad, Nuno Carvalhais, Martin Brandt, Rasmus Fensholt, Simon Besnard, Valerio Avitabile

**Affiliations:** Laboratoire des Sciences du Climat et de l’Environnement, LSCE/IPSL, CEA-CNRS-UVSQ, Université Paris Saclay, Gif-sur-Yvette 91191, France; Department of Geosciences and Natural Resource Management, University of Copenhagen, Copenhagen 1350, Denmark; Laboratoire des Sciences du Climat et de l’Environnement, LSCE/IPSL, CEA-CNRS-UVSQ, Université Paris Saclay, Gif-sur-Yvette 91191, France; Earth Observation for Ecosystem Management, School of Life Sciences, Technical University of Munich, Freising 85354, Germany; Gamma Remote Sensing, Gümligen 3073, Switzerland; Laboratoire des Sciences du Climat et de l’Environnement, LSCE/IPSL, CEA-CNRS-UVSQ, Université Paris Saclay, Gif-sur-Yvette 91191, France; Laboratoire des Sciences du Climat et de l’Environnement, LSCE/IPSL, CEA-CNRS-UVSQ, Université Paris Saclay, Gif-sur-Yvette 91191, France; Laboratoire des Sciences du Climat et de l’Environnement, LSCE/IPSL, CEA-CNRS-UVSQ, Université Paris Saclay, Gif-sur-Yvette 91191, France; Laboratoire des Sciences du Climat et de l’Environnement, LSCE/IPSL, CEA-CNRS-UVSQ, Université Paris Saclay, Gif-sur-Yvette 91191, France; Max Planck Institute for Biogeochemistry, Jena 07745, Germany; Department of Geosciences and Natural Resource Management, University of Copenhagen, Copenhagen 1350, Denmark; Department of Geosciences and Natural Resource Management, University of Copenhagen, Copenhagen 1350, Denmark; GFZ Helmholtz Centre for Geosciences, Potsdam 14473, Germany; Consultant for Engineering Ingegneria Informatica S.p.A., Rome 00144, Italy

**Keywords:** forest carbon sink, climate change mitigation, forest disturbances, forest biomass

## Abstract

Forests are carbon sinks essential for climate change mitigation. However, increased harvests and natural disturbances across Europe have recently challenged this role. To project the future carbon sink capacity of Europe’s forests, we integrated country reports from the United Nations Framework Convention on Climate Change with remote sensing maps of disturbances and above-ground biomass. Our model simulates biomass dynamics at 18 km resolution from 2010 to 2030, predicting a 39% decrease in the EU-27 forest carbon sink, driven by disturbances outpacing biomass recovery. Consequently, the 2030 forest carbon sink will fall 27% short of the estimated target consistent with EU-27 climate objectives. We demonstrate that the three billion trees initiative is insufficient for climate change mitigation and needs to be combined with a 28% reduction in forest harvests from 2025 to 2030 to meet these targets.

## INTRODUCTION

European forests have gradually recovered from major timber exploitation during and following the two World Wars [1]. Today, they cover one third of the continent and play a major role in reaching the 2030 carbon sink target set by the European Union (EU-27) for the land-use sector under the United Nations Framework Convention on Climate Change (UNFCCC) [2]. The forest carbon sink is defined in this study as the sum of net carbon stock changes in five pools: above-ground and below-ground biomass, soils, deadwood (including litter), and harvested wood products (HWPs). We estimate the 2030 forest carbon sink target for the EU-27 to be 392 MtCO₂eq/yr. This value corresponds to the average forest carbon sink over 2016–2018 based on UNFCCC data, consistent with the land-use sector target.

Europe’s forests are facing increasing pressure from timber harvest [3,4], as well as from natural disturbances such as wildfires, storms, bark beetle outbreaks [5], and drought and heatwave events [6]. These disturbances can interact and cascade, thereby increasing forest vulnerability [7]. Over the past three decades, the mortality of forest trees has almost doubled in Europe [8], raising concerns about the future resilience of forests to disturbances [9]. Country reports to the UNFCCC indicate a declining trend in the EU-27 forest carbon sink, which is projected to continue until 2030 based on evidence from large-scale simulations using a forestry carbon model (CBM) [10], statistical extrapolations of current trends [11], and land-climate models under different Representative Concentration Pathway scenarios [12]. This raises concerns about the capacity of forests to sustain their role as major carbon sinks in the future [13–15]. To address this situation, the EU-27 has launched an ambitious initiative to plant 3 billion trees by 2030 [16], yet only 38 million trees have been planted so far [17]. In parallel, most European countries have endorsed the Glasgow declaration, committing to halt and reverse forest loss by 2030 [18,19].

Reliable observations of forest resources are essential for assessing the trajectory of the forest carbon sink. National Forest Inventories (NFIs) routinely monitor forest wood stocks [20] with a typical revisit cycle of 5 years, which complicates the tracking of changes in forest growth or stocks. Furthermore, access to individual plot observations is restricted due to economic and legislative issues, particularly since 47% of forests are privately owned [3]. Alternatively, forest above-ground-biomass carbon (AGC) stocks can be estimated with remote sensing products, such as PlanetScope imagery [21] (30 m resolution, 2019) and CCI-ESA v5 [22] (100 m resolution, 2015–2021). These products provide consistent carbon stock data across Europe, but lack the ability to quantify loss due to disturbances. Instead, the occurrence of forest disturbances [23] and their attributed types [24] (fires, harvests, storms, including bark beetle outbreaks) are monitored with Landsat data (1986–2020, 30 m resolution). This allows for the estimation of trends in forest cover loss and the partitioning between harvests and natural disturbances, but remains limited in estimating the severity of the disturbance.

To address the sparsity of NFI data and the limitations of remote sensing products, this study develops a data-driven carbon model (DDCM v1.2, 18 km resolution) based on forest growth following disturbances, inspired by bookkeeping models used in the tropics [25,26]. We model the AGC of forested parcels as local sigmoid curves (hereafter ‘recovery curves’) that represent forest growth until a disturbance causes a sharp decrease, followed by subsequent regrowth. Only two free parameters (disturbance severity and maximum potential AGC) are tuned to match UNFCCC data (2010–2021) when aggregating all forested parcels across Europe. At an 18 km resolution, the model accounts for the partitioning between harvests and natural disturbances, net change in forest area, and trends in disturbance severity and return periods. Furthermore, it incorporates spatial heterogeneity in forest growth, assumes regrowth after each disturbance, and tracks carbon transfer between each pool, including temporary storage in HWPs and deadwood. Consequently, the model simulations reconstruct a forest carbon sink consistent with UNFCCC data while matching remote sensing AGC products that have been bias-corrected with regional NFI data [27].

**Figure 1.**
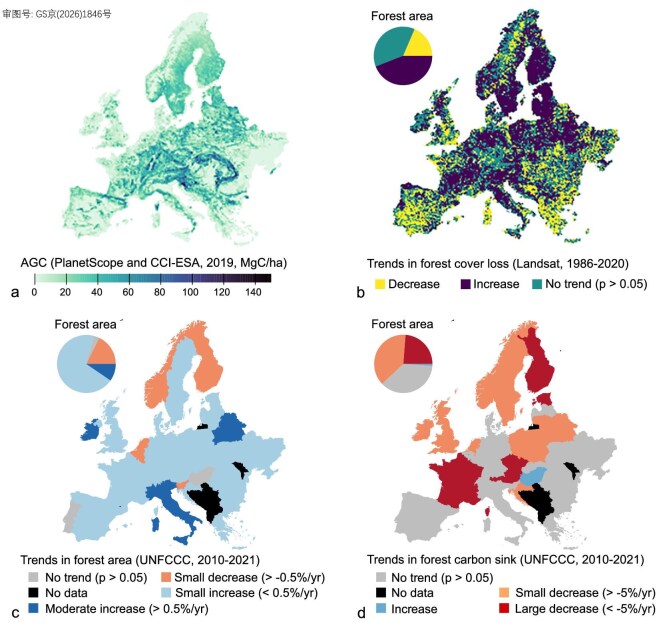
Forest state in Europe during the historical period. (a) Above-ground biomass for 2019, averaged from two products: PlanetScope [21] and CCI-ESA v5 [22], both bias-corrected with regional NFI data. (b) Trends in forest cover loss from 1986 to 2020 (Landsat [23]). (c) National trends in forest area from 2010 to 2021 (UNFCCC). (d) National trends in the forest carbon sink from 2010 to 2021 (UNFCCC).

**Figure 2.**
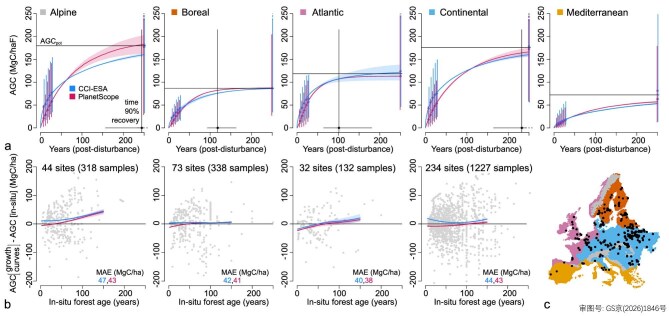
Forest recovery after disturbance. (a) Median of the recovery curves for five biogeographical regions. Black horizontal bars correspond to the potential AGC ($AG{C}_{pot}$). Black vertical bars correspond to the time required to recover 90% of $AG{C}_{pot}$. Whiskers correspond to the 95% range of values across all 18 km pixels. (b) Validation with age and AGC of trees from 383 sites in Europe (the Mediterranean region is omitted due to an insufficient sample size). (c) Locations of *in-situ* sites and biogeographical regions.

**Figure 3.**
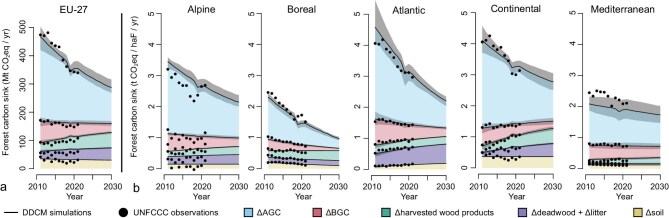
Projected decline in the forest carbon sink in a business-as-usual scenario. The forest carbon sink is defined as the sum of five components: net carbon stock change in AGC, BGC, HWPs, deadwood (including litter), and soils. (a) Forest carbon sink of the EU-27. (b) Forest carbon sink of the five biogeographical regions, normalized by forest area (in hectare of forests, haF) to facilitate comparison.

**Figure 4.**
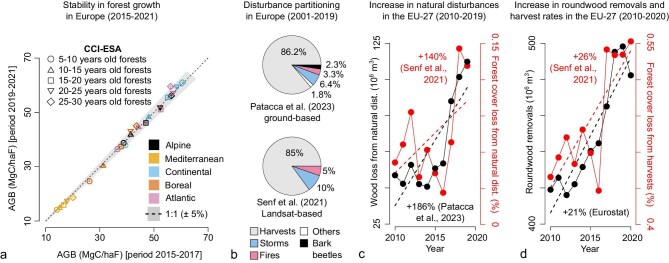
Drivers of the forest carbon sink decline. (a) Sensitivity test for changes in growth in forests of similar age between 2015–2017 and 2019–2021 (CCI-ESA). (b) Disturbance partitioning in Europe; note that bark beetles are grouped with storms in Landsat data. (c) Losses in wood volume (black) and forest cover (red) in the EU-27 due to natural disturbances. (d) Roundwood removals (black) and forest cover loss (red) in the EU-27 due to harvest or salvage logging. Panels (b), (c), and (d) integrate ground-based [5,39] and Landsat-based [23,24] observations.

**Figure 5.**
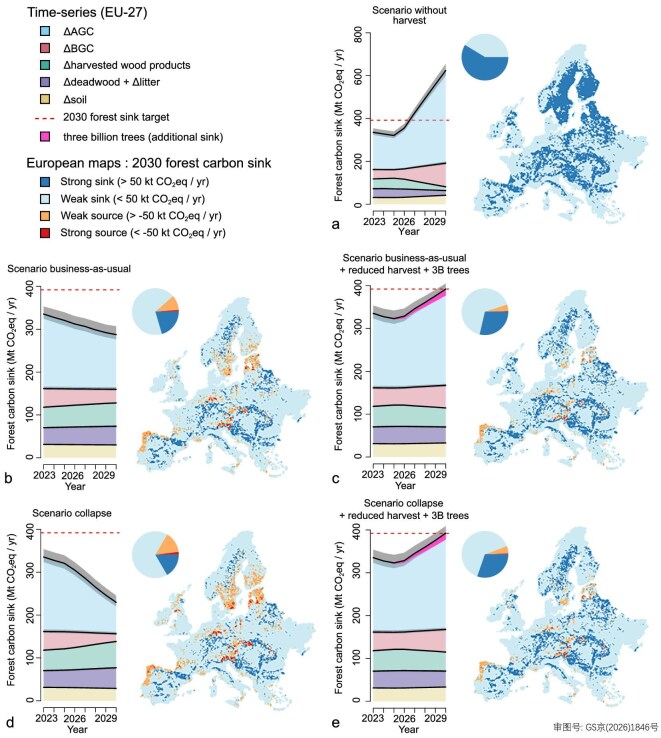
Future scenarios from 2025 to 2030. (a) ‘No harvest’ scenario. (b) Business-as-usual scenario. (c) Business-as-usual scenario with the necessary reduction in harvests and the three billion trees pledge to reach the 2030 forest carbon sink target. (d) ‘Collapse’ scenario: a 25% increase in natural disturbances and a 10% decrease in forest growth. (e) ‘Collapse’ scenario with the necessary reduction in harvests and the three billion trees pledge to reach the 2030 forest carbon sink target. Pie charts represent proportions relative to total forest area. Maps represent the 2030 forest carbon sink.

In the following, we first summarize state-of-the-art observations of AGC, forest cover loss, and national trends in forest area and forest carbon sinks (Fig. 1). We then introduce recovery curves (Fig. 2) to assess the potential recovery of Europe’s forests in the absence of disturbances. We present the projected decline in the forest carbon sink (Fig. 3) and explore its possible causes (Fig. 4). Finally, five scenarios for the trajectory of the forest carbon sink over the 2025–2030 period are explored (Fig. 5). The business-as-usual scenario assumes that forest growth and disturbance trends (including both harvest and natural disturbances) continue unaffected. The collapse scenario assumes that climatic stress and disturbance cascades reduce forest growth and increase the severity of natural disturbances. Two optimistic scenarios incorporate the EU-27 pledge to plant three billion trees by 2030 and quantify the harvest reductions necessary to reach the 2030 forest carbon sink target relative to the previous scenarios. The final scenario removes harvesting entirely to assess the potential forest carbon sink under a regime of pure natural disturbances.

## RESULTS AND DISCUSSION

### Current forest state in Europe

Europe held $12.09 \pm 0.04$ PgC of above-ground biomass carbon across $208.7$ Mha of forests in 2019 based on remote sensing products (CCI-ESA and PlanetScope) bias-corrected with NFI data (Fig. 1a). According to Landsat data, $44\% $ of these forest areas experienced increased forest cover loss from 1986 to 2020 due to disturbances (Fig. 1b). Despite this trend, forest area has been slightly increasing across the continent (Fig. 1c), reaching a net change of $+ 1.69\% $ from 2010 to 2021 (UNFCCC). These results are not contradictory: forest area definitions used in NFIs are based on land-use criteria, and disturbed forests flagged by Landsat are assumed to regenerate. However, annual summaries of country reports under the UNFCCC indicate that the carbon sink of $60\% $ of European forests has declined from 2010 to 2021 (Fig. 1d). Only Hungary and Liechtenstein have reported a significant increase in their national forest carbon sink.

### Forest recovery after disturbance

Forest recovery after disturbance shows significant variations across different biogeographical regions of Europe [28] (Fig. 2a). In the Boreal region, forests typically need 118 {93,163} years on average to regain 90% of their maximum potential AGC (AGC_pot_) after a stand-replacing disturbance event. The confidence intervals in brackets show the range obtained from model parameterization conducted on different AGC maps at different scales. Forests in the Atlantic also take about a century to recover (101 {64,181} years), while recovery in the Alpine and Continental regions is twice as slow (239 {157,312} years). Their recovery is slower because it is defined here as a percentage of the potential AGC (AGC_pot_), which is much higher in the Alpine and Continental regions (184 {162,223} MgC/haF, with haF standing for hectares of forest) compared to the Boreal and Atlantic regions (104 {85,147} MgC/haF). However, strong water limitation in the Mediterranean region results in the longest recovery time (more than 300 years) with the lowest potential AGC (72 {59,92} MgC/haF). While local recovery curves accurately match field observations on average (Fig. 2b), their precision is limited by environmental variability—such as climate and soil—and uncertainties in the field measurements themselves (Fig. S10).

On a global scale, studies have suggested that forests could significantly increase their biomass if allowed to regrow partially or fully [29–33]. Based on the recovery curves, we estimate that Europe could potentially increase its AGC stock by 15.8 {11.9,19.0} PgC in the absence of disturbances. This corresponds to a ∼130% increase in AGC carbon stocks relative to the period 2017–2020. This result is consistent with the conclusions of a study based on old-growth forests in Europe [34]. Being far from their saturation point, these forests reflect a broader shift in forest dynamics characterized by a rapid increase in forest turnover rates [4,35]. Mature forests steadily decline due to increased tree mortality, leading to significant ecological consequences [36].

### Decline in the forest carbon sink

According to the DDCM, the net carbon sink of EU-27 forests is projected to decrease from $467{\mathrm{\ }} \{ {435, 489} \} $ MtCO_2_eq/yr in 2010 to $287{\mathrm{\ }} \{ {276, 307} \}{\mathrm{\ }}$MtCO_2_eq/yr by 2030 in a business-as-usual scenario (BaU, Fig. 3a). We therefore predict a gap of $- 105{\mathrm{\ }}\{ { - 116, - 85} \}{\mathrm{\ }}$MtCO_2_eq/yr compared to our estimated 2030 target for the EU-27 (392 MtCO_2_eq/yr). The confidence intervals in brackets include both structural and parameter uncertainties through Monte-Carlo simulations (see Methods). The largest decrease in the forest carbon sink is expected in the Boreal region (Fig. 3b), with a decline of 58%, while the Mediterranean region is predicted to stay more stable, with a decrease of only 13%. Each component of the forest carbon sink has different significance and behavior. The net carbon stock change in living above- and below-ground biomass (ΔAGC + ΔBGC) will see a large decrease of 58% from 2010 to 2030 in the EU-27, which is the primary driver of the overall decline. However, this decline will be partially offset by increases in the net carbon stock changes of HWPs [11] (ΔHWP, +80%) and deadwood resulting from recent disturbances (ΔDWL, +63%). The net carbon stock change in soils is expected to decrease by 13%, consistent with our current understanding of the impact of harvests on soil carbon dynamics [37]. While HWPs and deadwoods only accounted for ∼12% of the EU-27 forest carbon sink in 2010, they are projected to contribute ∼34% by 2030, acting as a temporary buffer against the declining carbon sink of living biomass and soils. The BaU scenario forecasts a less optimistic carbon sink for living biomass (ΔAGC+ΔBGC) compared to former CBM predictions [10]: 159 MtCO_2_eq/yr (DDCM) vs. 240 MtCO_2_eq/yr (CBM) by 2030 for the EU-27. However, the CBM forestry model did not capture the recent declines in forest carbon sinks as reported in the UNFCCC data from 2023 [10].

We examine potential causes behind the decline in Europe’s forest carbon sink. Forest area has increased overall in Europe (Fig. 1c), indicating that deforestation is unlikely to be the dominant driver of the decline. In addition, natural saturation of the sink due to forest aging can be excluded, as forests are only at 43% of their potential biomass on average. Our remote sensing data analysis also suggests a relatively stable forest growth in Europe (within ±5%) from 2015 to 2021 (Fig. 4a). This aligns with a slight decrease in the gross productivity of forests in France (−4% from 2005–2013 to 2014–2022 [38]), which alone cannot explain the steep decline in its forest carbon sink (−64% from 2010 to 2021). The decline in Europe’s forest carbon sink is therefore more likely driven by disturbances, as reported from both ground-based and remote sensing estimations [5,23,24,39]. Timber harvest is by far the most significant disturbance (Fig. 4b), accounting for 85%–86% of all disturbances from 2001 to 2019, followed by storms (6%–7%), fires (3%–5%) and bark-beetles (∼3%). Over the 2010–2020 period, there is a large increase in mortality from natural disturbances (160% ± 25%, Fig. 4c) and a more moderate increase in forest loss from harvesting activities (20% ± 8%, Fig. 4d), consistent with the decline in the forest carbon sink. This increase in harvesting may reflect rising wood demand as well as salvage logging following natural disturbances, which is likely accounted for as roundwood removal [40].

### Future scenarios from 2025 to 2030

Harvesting plays a major role in the current forest carbon sink trajectory, as shown by the no-harvest scenario after 2025 (Fig. 5a). Eliminating harvesting would rapidly restore the sink to $624\ \{ {596 ,657} \}$ MtCO_2_eq/yr in 2030, well above the EU-27 2030 target (392 MtCO₂eq/yr). More realistically, a $28\ \{ {27,31} \}\ $% reduction in harvest rates from 2025 to 2030 would allow the EU-27 to meet the target relative to BaU (Fig. 5c). This includes the EU pledge to plant three billion trees, contributing about 15 MtCO₂eq/yr to the sink [41]. Contributions vary across regions, as do required harvest reductions (Table 1). The Atlantic region faces the greatest pressure ($40\ \{ {38,43} \}\ \% $ reduction needed in harvest rates), while the Boreal region is more on track ($27\ \{ {26,28} \}\ \% $). The ‘collapse’ scenario reflects a possible cascade of disturbances and higher climatic stress. In this case, harvests in the EU-27 would need to decrease by $49\ \{ {47,50} \}\ $% to meet the target. This first-order estimate should be refined by considering additional management options beyond harvest reduction [10,42], such as thinning, rotation changes, biodiversity-oriented regeneration, or non-intervention versus salvage logging [40]. Such options are essential, as a continent-wide harvest reduction may increase carbon leakage outside Europe to meet European demand.

**Table 1.** Projected forest area and carbon sink in Europe (2025–2030).

		Forest area (Mha)			Business-as-usual scenario		Collapse scenario	
	Forest carbon sink (MtCO_2_eq/yr)			Forest carbon sink target (MtCO_2_eq/yr)	Gap-to-target (MtCO_2_eq/yr)	Required harvest reduction (%)	Gap-to-target (MtCO_2_eq/yr)	Required harvest reduction (%)
Regions	2025	2025	2030	2030	2030	2025–2030	2030	2025–2030
Atlantic	55 [52,58]	21.1 [21.0,21.1]	21.2 [21.1,21.3]	75	−28 [−31, −26]	40 [38,43]	−38 [−40, −35]	51 [47,55]
Alpine	71 [67,77]	29.8 [29.7,29.9]	29.9 [29.8,30.1]	86	−22 [−25, −16]	36 [34,38]	−33 [−37, −28]	53 [52,55]
Boreal	79 [73,82]	64.4 [64.2,64.5]	64.6 [64.3,64.8]	113	−51 [−53, −48]	27 [26,28]	−73 [−75, −71]	46 [44,48]
Contin.	188 [180 197]	62.6 [62.4,62.7]	63.1 [63.0,63.3]	218	−43 [−52, −34]	28 [23,33]	−65 [−74, −57]	55 [54,56]
Mediter.	61 [54,69]	33.5 [33.4,33.6]	33.9 [33.8,34.1]	64	−5 [−12,+2]	29 [27,30]	−12 [−18, −5]	46 [45,48]
EU-27	321 [309,340]	164.2 [163.8,164.5]	165.2 [164.8,165.6]	392	−105 [−116, −85]	28 [27,31]	−162 [−173, −147]	49 [47,50]

Data are presented by five biogeographical regions and EU-27. Harvest reduction required to meet 2030 targets (including the three billion trees pledge) is estimated under two scenarios: business-as-usual and ecosystem collapse.

The resolution of the DDCM allows for the detailed computation of spatial variations in the forest carbon sink in 2030 (maps in Fig. 5). Regions such as Southern Germany, Northern Belarus, Northern Sweden, Southern Romania, Central Spain, the Pyrenees, and the Dinaric mountains are expected to remain large forest carbon sinks. These sinks are attributed to either stationary or decreasing trends in disturbance levels (partly due to recovery from old disturbances like in Southern Germany), unlike other parts of the continent (Fig. 1b). Particularly, forests in Portugal, Slovenia, Southern France, Austria, Czechia, Estonia, Latvia, Northern Germany, southern Sweden, and southern Finland are projected to face severe declines in their respective carbon sinks. Between $13\ \{ {11,16} \}\ \% \ $and $18\ \{ {16,21} \}\ \% \ $of European forests are expected to become net carbon sources in 2030 depending on the scenarios (BaU vs. collapse, respectively). A reduction in harvest rates would have the greatest impact in central and northern Europe and could help offset the dramatic increase in natural disturbances.

### Challenges and mitigation strategies

A common assumption is that sustainable harvests carry a low carbon cost according to the rationale that (i) HWPs serve as a reliable long-term carbon sequestration asset, and (ii) young stands replacing mature forests are compensating for the carbon deficit because they are more productive [43]. Based on these premises, the EU-27 has endorsed wood use for bioenergy, which now represents 59% of renewable energy [44]. Regarding point (i), 69% of global HWPs have very short lifespans, significantly reducing their contribution to the forest carbon sink [43]. For instance, estimates from the French NFI show that 68% of HWPs are used as bioenergy [45]. Concerning point (ii), the DDCM demonstrates that the recovery of young and productive stands is insufficient to offset carbon losses from current natural and anthropogenic disturbances (Fig. 5b). These projections also address point (i) as they implicitly incorporate carbon transfers across different pools (especially HWPs and deadwood). The EU-27 plan could be made more effective by extending HWP lifespans (e.g. using wood for construction material) and reducing wood use for bioenergy (by promoting other renewable energies) to lower harvest rates, especially as natural disturbances continue to increase dramatically (Fig. 4c).

Several limitations are acknowledged in our study. First, carbon gains or losses associated with land-use change are not explicitly modeled, but are instead implicitly captured through changes in net forest area. This is a reasonable simplification given the relative stability of forest cover in Europe. Second, we did not incorporate the concept of ‘avoided emissions’ into the EU-27 target. Decisions to reduce deforestation and degradation locally (e.g. under the Glasgow Declaration) may influence national sink target calculations. In Europe, this effect is moderate and mainly concerns Ireland, the UK, northern Spain, and parts of the eastern Balkans [19]. Third, gap-to-target estimates rely on a 2016–2018 baseline, consistent with the EU-27 methodology. While this correctly captures the target at the continental scale (Fig. S7), country-specific adjustments are not accounted for. Therefore, results are presented at the level of biogeographical regions (Table 1) rather than individual countries. Finally, disturbance type and severity (e.g. fires, storms, and harvesting), along with patch size and forest structure (e.g. stand age, species type, and plantations vs. natural forests), may influence recovery dynamics [46]. We will address these factors and their legacy effects in future work.

We present evidence that Europe’s forests are increasingly at risk of losing their role as carbon sinks, primarily due to a dramatic increase in natural disturbances alongside a moderate increase in harvests. While these forests have the potential to double their AGC stocks, $13\% {\mathrm{\ to\ }}$$18$% of European forests are projected to become a net carbon source in 2030, as intensifying disturbance regimes exceed recovery rates. The carbon sequestration capacity of the remaining forests is progressively deteriorating, a trend exacerbated by business-as-usual forest management practices. Projections for the near future are less optimistic than our estimated target for the EU-27, which will significantly impact European climate change mitigation plans that rely on increasing forest area. Over the past two decades, forest expansion has been limited. Even if the 3 billion trees pledge was met, we estimate that a $28\ \{ {27,31} \}\ \% $ decrease in forest harvest from 2025 to 2030 would be required to reach the target. Historically, forest management in Europe has accumulated a substantial carbon debt [47], further exacerbated by recent natural disturbances. To mitigate the decline in the European carbon sink, a major shift in forest management practices is essential for facing the dramatic increase in natural disturbances.

## METHODS

The data-driven carbon model (DDCM version 1.2, Figs S1 and S2) simulates annual AGC stocks at 18 km resolution. The net carbon stock change in AGC for a year *y* is then computed as ${\mathrm{\Delta }}AGC( y ) = AGC( y ) - AGC( {y - 1} )$. The net carbon stock changes in belowground biomass (${\mathrm{\Delta }}BGC$), soils (${\mathrm{\Delta }}S)$, harvested wood products (${\mathrm{\Delta }}HWP)$, and deadwood (including litter, ${\mathrm{\Delta }}DWL$) are estimated based on${\mathrm{\ \Delta }}AGC$ with linear relationships derived from UNFCCC data in five different biogeographical regions (Fig. S4). This provides the annual forest carbon sink at 18 km, as defined $FS( y ) = {\mathrm{\Delta }}AGC( y ) + {\mathrm{\Delta }}BGC( y ) + {\mathrm{\Delta }}S( y ) + {\mathrm{\Delta }}HWP( y ) + {\mathrm{\Delta }}DWL( y )$.

The $AGC( y )$ is computed from the imbalance between AGC loss due to disturbances and AGC gain due to forest growth or recovery from disturbances. The annual percentage of AGC loss $s( y )$ is modeled at 18 km resolution with a linear relationship derived from 30 m forest cover loss data [23]. AGC gains are modeled at 18 km resolution with a Chapman–Richards function (Fig. 2a): $AGC( t ) = \ AG{C}_{pot}{( {1 - {e}^{ - bt}} )}^c$ that requires three parameters $b,c$, and $AG{C}_{pot}$. The potential AGC ($AG{C}_{pot}$) is estimated with the AGC of forests undisturbed since 1986. The two other coefficients $b$ and $c$ are retrieved with a space-for-time analysis (Fig. S6). The *in-situ* AGC and age of NFI data validating our recovery curves (Fig. 2b) originate from a diverse forest inventory collection previously used to upscale forest age [48] and can be accessed upon request. Two regional upscaling factors $\alpha $ and $\beta $ (free parameters) are then applied to $s( y )$ and $AG{C}_{pot}$, respectively, so that DDCM simulations match the UNFCCC observations (2010–2021, Fig. S5). The reduction in harvest necessary for the EU-27 to meet the estimated target by 2030 has been simulated starting in 2025, knowing the ratio at 18 km between harvests and natural disturbances provided by the disturbance map (Figs S13 and S14).

In particular, version 1.2 of the DDCM (i) matches the AGC of European countries based on NFI data for the year 2020 (Fig. S3), (ii) accounts for changes in the net forest area (based on national trends from UNFCCC), (iii) captures long-term trends (30-year periods) at 18 km in natural disturbances and harvests, and finally (iv) ignores the AGC changes according to the maps from CCI-ESA (2015–2021, these are contaminated with artefacts, Fig. S17). A single year of AGC data (2019 for PlanetScope and mean of 2017–2021 for CCI-ESA) is used to run the DDCM from 2010 to 2030. As a proof of concept, Fig. S16 illustrates the DDCM simulation parameterized using only the UNFCCC data from 2010. Despite no prior information about the decline in the forest carbon sink from 2011 to 2021, the model accurately replicates this trend. This outcome demonstrates that the model avoids overfitting the UNFCCC data and effectively captures the key drivers of the forest carbon sink decline: increasing rates of natural and anthropogenic disturbances, coupled with insufficient compensation from deadwood and HWP sinks, despite their growth.

### Forest carbon sink target

The EU-27 defined the LULUCF sink target as 310 MtCO_2_eq/yr in 2030 [2]. The LULUCF sink is the sum of multiple components: forest lands, HWPs, croplands, grasslands, wetlands, settlements, and ‘other’ lands. Their target has been partly established on the average greenhouse gas inventory data for the years 2016, 2017 and 2018 [2]. We therefore recalculated the target for the forest carbon sink only (net carbon stock change in AGC, BGC, deadwood and litter, soil, and HWPs) of all EU-27 countries from 2016 to 2018 based on the UNFCCC reports. This led to the value of 392 MtCO_2_eq/yr, defined as the 2030 forest carbon sink target in our study and consistent with the broader LULUCF sink target (Fig. S7).

## Supplementary Material

nwag354_Supplemental_File

## Data Availability

The data used in this study are permanently and publicly available on a Zenodo repository (https://doi.org/10.5281/zenodo.20203371). The raw *in-situ* data are available upon request (ncarvalhais@bgc-jena.mpg.de). The code in R and Python used in this study is permanently and publicly available on a Zenodo repository (https://doi.org/10.5281/zenodo.20203593).
