# Supplementary material for: Alarming decline in the carbon sink of European forests driven by disturbances
Source: Natl Sci Rev. 2026 Jun 11;13(15):nwag354. doi: 10.1093/nsr/nwag354 (PMC13463641; doi:10.1093/nsr/nwag354)
Supplement: Supplementary file 1 [file nwag354_supplemental_file.docx]

*Supplement of* **“Alarming decline in the carbon sink of European forests driven by disturbances”**

**Extended Methods**

**Biogeographical regions.** The biogeographical regions (Fig. 2c) are defined by the European Environment Agency (https://www.eea.europa.eu/en). Some regions (Arctic, Pannonian, Anatolian, Macaronesia, Steppic and Black Sea) are located beyond the study’s boundaries and merged with their closest neighbor (arctic with boreal, the remaining regions with continental). Europe is therefore divided into five biogeographical regions with different characteristics based on topography (the Alpine region includes all major mountains) or climate (Boreal, Atlantic, Continental, Mediterranean). **Forest mask.** This study exclusively focuses on forest lands based on the harmonized NFI dataset on a sub-national scale across Europe for the year 2020 [1]. As Ukraine, Moldova and Belarus were missing in this dataset, we extended it using the forest area documented for these countries in the 2020 reports from the Food and Agriculture Organization of the United Nations, Global Forest Resources Assessment (FAO-FRA [2]). We created a gridded forest mask at 30 m in Europe (EPSG:3035) that matches the NFI forest area with the following procedure. We first calculated the mean of two existing forest products at 30 m: the forest mask from the 1986-2020 disturbance map [3] (discrete values of 0 or 1) and the forest canopy cover from the 2019 PlanetScope map [4] (continuous values between 0 and 1). Then, for each departmental unit in Europe (288 units distributed among 40 countries), we calculated a threshold above which the aggregated sum of our forest mask corresponds to the NFI forest area for this unit (Fig. S3). All 30 m pixels above this threshold were set to 1, otherwise 0. We did not encounter major problems where NFIs reported a forest cover much higher than the mean of the two forest masks. The mean threshold across all units (288) is 0.59 $\pm$0.26 ($\pm$ 1 SD).

**AGC maps.** The annual AGC CCI-ESA maps [5] (2015 to 2021, version 5, at 100 m resolution) and the 2019 PlanetScope-based AGC map [4] (30 m, version 0.1) are downscaled with a bilinear resampling to the 30 m forest mask, and non-forested pixels are discarded. The 2010 CCI-ESA AGC map is discarded because of known biases due to the sensor difference (Fig. S8B). All AGC maps have been bias-corrected to match the AGC reported at the sub-national scale in the harmonized NFI dataset for the year 2020 [1]. As Ukraine, Moldova and Belarus were missing in the harmonized NFI dataset, we extended it using the AGC documented for these countries in the FAO-FRA reports [2] for 2020. The bias correction for each European departmental unit is a multiplicative factor corresponding to the ratio between the AGC reported by NFIs and the mean raw AGC of 2020 for CCI-ESA or 2019 for PlanetScope (Fig. S3). The median correction across all units (288) is 1.30 $\pm$0.27 ($\pm$ 1 Mean Absolute Deviation) for CCI-ESA, and 1.06 $\pm$0.24 ($\pm$ 1 MAD) for PlanetScope.

**UNFCCC reports.** We used the 2023 common reporting format reports (CRF) of all 33 countries available in Europe (<https://unfccc.int/ghg-inventories-annex-i-parties/2023>) for the years 2010 to 2021. For each country and each year, we extracted the following components from Table 4a (row “A. total forest land”): “Total area (kha)”, “net carbon stock change in living biomass (ktC)”, “Net carbon stock change in dead wood (ktC)”, “Net carbon stock change in litter (ktC)”, “Net carbon stock change in mineral soils (ktC)” and “Net carbon stock change in organic soils (ktC)”. We extracted the following components from Table 4: “4. Total LULUCF (Land Use, Land-use Change and Forestry) for the Net CO2 emissions/removals (ktCO2)” and “G. Harvested wood products for the Net CO2 emissions/removals (ktCO2)” (ΔHWP). For each year and each country, we converted all components in MgC/haF (Megagrams of Carbon per hectare of forests) by dividing them by the forest area (and multiplying the components in ktCO2 by 12/44 to obtain ktC). Two obvious errors have been found and corrected in the UNFCCC report of two countries (Fig. S8): (i) France reported a forest area including its overseas territories, but the forest sink has been computed across metropolitan France only (this error has been corrected using an official report from the International Technical Centre on Air Pollution and Climate Change, CITEPA [6]), (ii) The detailed forest sink reported by Portugal in Table 4a does not correspond to the forest sink used in their LULUCF report in Table 4 (We used the forest sink from Table 4a and updated their LULUCF budget). For all countries, we partitioned the “net change in carbon stock change in living biomass” (ΔLB) into two new components: “net change in carbon stock change in above-ground biomass” (ΔAGC) and “net change in carbon stock change in below-ground biomass” (ΔBGC). This partitioning has been performed using $\Delta AGC=\frac{1}{1+\varepsilon}\Delta LB$ and $\Delta BGC=\frac{\varepsilon}{1+\varepsilon}\Delta LB$ with $\varepsilon=\frac{BGC}{AGC}$. The factor $\varepsilon$ has been calculated for each country (Fig. S4A) based on the national AGC and BGC documented in the FAO-FRA reports [2] for 2020 (The year 2015 has been used instead for Serbia, Albania, Cyprus, Portugal, Greece and Malta as the year 2020 was not available yet). The mean $\varepsilon$ across all countries is 0.25 $\pm$0.06 ($\pm$ 1 SD). To reduce the large discrepancies between country reports, we summed the “Net carbon stock change in organic soils” with “Net carbon stock change in mineral soils” to create a single component “Net carbon stock change in soils (ΔSoil)”. Similarly, we summed the “Net carbon stock change in deadwood” with “Net carbon stock change in litter” to create a single component “Net carbon stock change in deadwood and litter (ΔDWL)”. For each year $y$ and each country $c$, the forest sink is therefore defined in this study as the sum of five components: ${FS}_{c}(y)={\Delta AGC}_{c}(y)+{\Delta BGC}_{c}(y)+{\Delta Soils}_{c}(y)+{\Delta DWL}_{c}(y)+{\Delta HWP}_{c}(y)$ expressed in MgC/haF. For each biogeographical region, we reconstructed the five components of the forest sink based on each country's forest area overlapping with the biogeographical region (according to our forest mask, Fig. S4B). Let *b* be a biogeographical region, ΔV one of the five components, and $c$ a country with an overlap of forest area ${FA}_{c}$ with *b* (${FA}_{c}=0$ if the country is outside the biogeographical region), then: ${\Delta V}_{b}=(\sum_{c} {FA}_{c}{\Delta V}_{c})/\sum_{c} {FA}_{c}$ (in MgC/haF). For each biogeographical region, linear relationships of ${\Delta V}_{b}$ versus ${\Delta AGC}_{b}$ were calculated (the mean is computed instead if the trend is non-significant, p > 0.05) based on 12 data points (2010 to 2021). The linear model is justified here because the forest sink ${FS}_{b}$ is strongly linearly related to ${\Delta AGC}_{b}$, with a $r^{2}$ varying from 0.87 to 0.99 for the five biogeographical regions. These linear relationships were used in the DDCM model to estimate each forest sink component at 18 km from 2010 to 2030 (Fig. S4D-G). Hungary is the only country reporting an increasing trend in their forest sink, which we currently do not explain. The growth rate or the disturbance proportions of Hungary do not particularly show large differences compared to its neighbors, Fig. S11.

**Disturbances.** Disturbances are defined in this study at 18 km resolution based on spatial and temporal aggregations of the severity in the forest cover loss at 30 m (Landsat-based [3]). These disturbances aggregated at 18 km are associated with two annual characteristics: (i) $s\left( y \right)$, the annual percentage of AGC loss with *y* a year, and (ii) $\tau\left( y \right)$, the return interval (which is the average time between two mean disturbances at 18 km across 30 years). We explain how we estimated them in the following. For each year (1986 to 2020), the 30 m severity values are summed in 18 km pixels and divided by the forest area to produce a time series of the percentage of forest cover loss in each year. For each 18 km pixel, these annual percentages are averaged over six 30-year windows (1986-2015 to 1991-2021) and a linear trend ($f_{loss}(y)$) is calculated through the six mean values to extrapolate the percentage of cover loss from 2010 to 2030 (the mean is computed instead if p > 0.05). The conversion from a percentage of cover loss to a percentage of AGC loss is given by $s\left( y \right)=\beta f_{loss}(y)$, with $\beta$ a correction factor calculated on the scale of a country or a biogeographical region (section ”DDCM parameterization”). For each 18 km pixel and six 30-year windows (from 1986-2015 to 1991-2020), the return interval of the mean disturbance is characterized by $\tau=\frac{1}{P}$ with $P$ the exceedance probability compared to the mean percentage of forest cover loss in this pixel. $P$ is estimated [7] with the number of years associated with a forest cover loss above the mean loss (across 30 years at 18 km) divided by the time-series length (30 years). A linear trend is calculated based on the six computed values$\tau$ per pixel to extrapolate $\tau$ from 2010 to 2030. If the trend is not significant (p > 0.05), the median is computed instead because $\tau$ takes discrete values. Based on 2005 values, the Atlantic and Continental regions share similar return intervals (~3$.3\pm0.1$ years), the Alpine and Mediterranean have higher return intervals (~3$.9\pm0.2$ years), and the Boreal has lower return intervals (2.7 years). The trends are non-significant (p > 0.05) for ~67% of European forests, without obvious spatial patterns for the remaining forests (Fig. S9). It is important to notice that this return interval does not refer to disturbance events at 30 m (which occur much more rarely) but to disturbances aggregated at 18 km. Finally, we shortly discuss uncertainties related to the Landsat-based disturbance map at 30 m [3]. This product relies on the best available pixel composites; therefore, data density affected by changes in technological advances in Landsat has a rather marginal effect. The accuracy has probably not significantly shifted as all data processed to level-2 have the same quality standards, and the spectral differences between different sensors (TM, ETM+ and OLI) have been polished. Finally, the LandTrendr algorithms used to detect a disturbance event [3] remove a lot of noise, resulting in a relatively high omission error. Hence, the overall disturbance rates are likely underestimated.

**Partitioning between natural disturbances and harvests**. The partitioning between natural disturbances and harvests has been obtained at 18 km (Fig. S13) based on the disturbance attribution to three agents using Landsat data at 30 m [8]: fire, storms (including bark-beetles that represent less than 3% of the total disturbances according to [9]) and harvests (including “others” that represent less than 2%). For each agent and each year (1986-2020), we spatially summed their associated severity (spectral indice between 0 and 1) from 30 m to 18 km. To flag and discard suspicious pixels at 18 km, we compared the Landsat-based harvests map with a wood production map constrained by NFI data from 2000 to 2010 [10]. To do so, we produced two maps at 18 km: (i) the total sum of severities associated with harvests from 2000 to 2010 at 18 km (Landsat-based map), and (ii) the total sum of wood production from 2000 to 2010 at 18 km (NFI-based map). Because these two maps use two different units, we computed a quantile version of each map with discrete values of 0, 0.1, 0.2,…, 1 (Fig. S12). A value of 1 means a hotspot of harvest (or wood production) compared to the rest of the continent. We calculated the absolute difference between the two quantile maps. We found 61% of pixels with high agreement (absolute difference below 0.1), 25% of pixels with moderate agreement (absolute difference between 0.2 and 0.3 of difference) and 14% of pixels with low agreement (absolute difference above 0.4). We discarded the pixels of low agreement from the entire study (especially for Fig. 4b described below and for Fig. 4c and Fig. 4d described in the section “recent increase in natural disturbances and harvests”). To produce the map of the ratio between natural disturbances and harvests at 18 km, we first computed the sum of severities associated with natural disturbances (fires and storms) from 1986 to 2020 at 18 km, and then we computed the sum of the severities associated with harvests from 1986 to 2020 at 18 km. We calculated the ratio $R$by dividing both maps at 18 km. We imputed the 14% missing pixels associated with a low agreement with the wood production map. To do so, we first flagged pixels that (i) share similar wood production with the missing pixels (low/medium/high based on the terciles threshold of the percentile map, i.e. 0.33 and 0.66), and (ii) are associated with a high agreement with the wood production map (i.e., their Landsat-based disturbance attributions are trustworthy). We replaced the ratio of the missing pixels with the median ratio from these three groups (low/medium/high wood production, see Fig. S13). We performed a spatial aggregation of the remaining missing pixels in Europe. Finally, as Ukraine was missing, we gave this country the median ratio value across Europe (17%). In parallel, we also tested for a possible change through time of the ratio between natural disturbances and harvests across Europe (Fig. S14). We aggregated the total sum of severities (from the Landsat map) associated with natural disturbances for each year. We divided them by the total sum of severities associated with harvests for each year (we discarded the low-confidence pixels from Fig. S12). We did not detect a significant trend (p = 0.14 > 0.05) in Europe’s ratio between natural disturbances and harvests from 1986 to 2020, which confirms earlier ground-based reports of natural disturbances [9]. The mean value is 17%, which is remarkably close to to the ground-based continental value of 16% [9]. We finally describe here the design of Fig. 4b. The Landsat-based partitioning in Fig. 1C has been computed using the total sum of severities from 2001 to 2019 associated with the three main agents: fires, storms (including bark-beetles) and harvests (including “others”). Low-confidence pixels at 18 km (described previously) were discarded beforehand. The ground-based partitioning in Fig. 4b was first established between harvests and natural disturbances following numbers given in [9] (period 2001-2019, natural disturbances accounted for 16% of the mean annual harvest in Europe), then the natural disturbances have been further partitioned into fires, storms, bark-beetles and “others” with their estimates corresponding to the period 1950-2019.

**AGC recovery curves.** The AGC of forests recovering after a stand-replacing disturbance (Fig. 2a) is reconstructed at the scale of small regions (18 km grid cells) to account for the spatial variability that exists in their biotic and abiotic drivers (in soil properties, tree species or climates [11])*,* and also to capture a sufficient number of 30 m stand-replacing disturbances in each 18 km pixel (10% of the forest area of each pixel has been disturbed on average from 1986 to 2020). The year of the disturbance is first subtracted from the year of the AGC map to obtain post-disturbance years. The AGC maps are then normalized by dividing them with ${AGC}_{pot}$ defined here as ${AGC}_{pot}= \alpha{AGC}_{75\%}$, with ${AGC}_{75\%}$ the 75% percentile of the AGC of all 30 m forests undisturbed since 1986 inside an 18 km pixel, and $\alpha$ a correction factor calculated on the scale of a country or biogeographical region (section ”DDCM parameterization”). For each normalized AGC map (2015-2021 for CCI-ESA and 2019 for PlanetScope) and each 18 km pixel, five mean AGCs are calculated within five-time windows ([5-10 years], [10-15 years],..,[25-30 years]). For each time window, the average of the seven products of CCI-ESA (2015-2021) is computed to reduce uncertainties. A Chapman-Richard recovery curve (${AGC}_{CR}\left( t \right)= {(1-e^{-bt})}^{c}$) is fitted through the five values to estimate the $b$ and $c$ coefficients. Recovery curves with non-physical inputs are discarded (AGC[5-10 years] > AGC[25-30 years]), and outliers in $b$ and $c$ populations across Europe are removed using the Logbox method [12]. Recovery curves associated with $r^{2}<0.5$ are finally discarded (Fig. S6). To visualize the representative AGC recovery curve for a given biogeographical region (Fig. 2a), we first normalized each curve (divided them by their ${AGC}_{pot}$ at 18 km), then stacked them by calculating the median for the age varying from 0 to 250 years. We finally multiplied this representative curve by the median ${AGC}_{pot}$ of all 18 km pixels with valid recovery curves. The time $t_{90}$ required to recover 90% of the ${AGC}_{pot}$ after a stand-replacing disturbance corresponds to ${AGC}_{CR}\left( t_{90} \right)=0.9{AGC}_{pot}$ which leads to $t_{90}=-\frac{1}{b}log(1-{0.9}^{\frac{1}{c}})$. The median $t_{90}$ has been calculated from all 18 km pixels with valid recovery curves. This procedure has been repeated for each AGC map (AGC maps from CCI-ESA have been averaged from 2017 to 2020) and each model parameterization (see “DDCM parameterization”) to estimate the uncertainty.

**Forest area trends.** To account for long-term changes in net forest area, we estimated the annual growth rate of forest area at a spatial resolution of 18 km based on national inventories reported in the UNFCCC from 2010 to 2021. We also extended the national forest area timeseries to each biogeographical region (see section UNFCCC reports). For each reporting unit (country or biogeographical region), an annual growth rate (r) was calculated as the linear slope of the relative change in forest area (A) between 2010 and 2021, normalized by the 2010 baseline: $r= \frac{A_{2021}-A_{2010}}{11\times A_{2010}}$. This rate represents the fractional annual change in forest area relative to the base year. The annual slope of forest cover change at the pixel level was then derived by multiplying the initial forest cover fraction by the assigned growth rate at the country or biogeographical region level. Finally, we calculated the intercept of the forest cover trend relative to the year 2019 to ensure temporal consistency with the reference AGC maps used in this study. This procedure resulted in two sets of spatialized forest cover trends — one constrained by country-level inventories and another by biogeographical regional aggregates — both provided in fractional units (0–1). These maps allow the DDCM to simulate the net expansion or contraction of forest area across the European continent while remaining consistent with official ground-based reporting.

**DDCM.** For each 18 km pixel, the AGC value from the PlanetScope map (2019) or CCI-ESA maps (averaged from 2017 to 2021 to center it around 2019) is the starting point for a backward simulation (from 2019 to 2009) and forward simulation (from 2019 to 2030) of AGC. Each simulation is the mean of 50 replicates associated with a different starting date for the first disturbance. One replicate simulates successive periods of growth $\tau(y)$ (the return interval of the mean disturbance at 18 km for a year $y$) based on ${AGC}_{CR}\left( t \right)= {{AGC}_{pot}(1-e^{-bt})}^{c}$, followed by a disturbance of severity $\tau(y)\times s(y)$, with $s(y)$ the annual percentage of AGC loss for the year $y$. Once the simulated AGC maps are produced from $y=2009$ to $y=2030$, the annual changes in AGC is calculated: $\Delta AGC\left( y \right)=AGC\left( y \right)-AGC(y-1)$. Knowing the linear relationships between the forest sink components and observed $\Delta AGC$ for the five biogeographical regions (section “UNFCCC”, see Fig. S4D-G), the forest sink is reconstructed at 18 km across Europe with projections from 2010 to 2030 (Fig. 3,5). The outputs from the DDCM at 18 km are therefore: linear coefficients of the trend in the forest fraction ($f_{c}\left( y \right)=a_{fc}y+b_{fc}$, unitless), forest age, linear coefficients of the trend in the percentage of AGC loss ($s\left( y \right)=a_{s}y+b_{s}$ in annual % of AGC loss), linear coefficients of the trend in the disturbance return interval ($\tau\left( y \right)=a_{\tau}y+b_{\tau}$ in years), potential AGC (${AGC}_{pot}$ in MgC/haF), recovery curves coefficients ($b$ and $c$, unitless), simulated AGC from 2009 to 2030 (in MgC/haF), and the forest sink components from 2010 to 2030: $\Delta AGC, \Delta BGC, \Delta HWP, \Delta DWL \mathrm{and} \Delta Soil$(net carbon stock change in MgC/haF). Malta and Cyprus are located outside of our dataset boundary; however, they represent only 0.03% of the European forest sink. Therefore, we simulated their sink component from 2010 to 2030 with a linear extrapolation of their current trends. Any missing values (in the inputs or outputs of the DDCM) at 18 km are spatially interpolated across Europe, with the percentage of missing values given in the supplementary (Fig. S8).

**DDCM parameterization.** AGC simulations from the DDCM model are sensitive to two parameters at 18 km: (i) the potential AGC (${AGC}_{pot}$), which is the maximum reachable AGC for a forest left undisturbed during a long period of time, and (ii) the percentage of AGC loss due to disturbances ($s(y)$). These two parameters remain difficult to estimate because (i) old-growth forests are scarce in Europe and undisturbed forests can be traced back to 1986 only (Landsat range), and (ii) the disturbance map built on Landsat images captures the forest cover loss only (and not the AGC loss). To solve this issue, we introduced two corrections $\alpha$ and $\beta$ (Fig. S5) such as: ${AGC}_{pot}= \alpha{AGC}_{75\%}$ and $s\left( y \right)=\beta f_{loss}(y)$. The variable ${AGC}_{75\%}$ is the 75% percentile of the AGC of all 30 m forests undisturbed since 1986 inside an 18 km pixel and $f_{loss}(y)$ is the linear trend at 18 km in the percentage of forest cover loss based on six 30 years moving averages of Landsat data (see section “disturbances”). For each AGC dataset (average of AGC maps from 2017-2021 for CCI-ESA, or PlanetScope map of 2019), values of ($\alpha$,$\beta)$ have been estimated on a country-level or a biogeographical region-level using a bootstrap analysis that minimizes the RMSE between DDCM simulations and observations of $\Delta$AGC from UNFCCC reports across the recent historical period (2010-2021). Then these ($\alpha$,$\beta)$ correction maps have been smoothed at 100 km to reduce the discrepancies at the border between two countries or two biogeographical regions. There are therefore four different versions of the outputs of the DDCM model: CCI-ESA (parameterization conducted on each country), CCI-ESA (parameterization conducted on each biogeographical region), PlanetScope (parameterization conducted on each country) and PlanetScope (parameterization conducted on each biogeographical region). Each version has different AGC recovery curves, disturbance trends and projected forest sinks.

**Uncertainty analysis.** We assessed the robustness of our carbon sink estimates by propagating uncertainties through a Monte Carlo framework. To quantify the spatial variability and confidence of our input parameters, we first conducted a sensitivity analysis. For a given input parameter P at 18 km (disturbance severity $s$, return interval $\tau$, growth coefficients $b$ and $c$, disturbance partitioning $R$, coefficients $a_{fc}$ and $b_{fc}$ of the net forest area trends), we extracted 9 values within a moving window ($54$ km) and fitted a local linear trend to produce residuals that account for environmental gradients. The local uncertainty was defined as the Median Absolute Deviation (MAD) of these residuals normalized by the local median (Fig. S18). Based on the distribution of these uncertainties across Europe, we categorized each 18 km pixel associated into three variability tiers for each P: high, medium and low variability (Fig. S19). We then conducted 1000 Monte Carlo simulations (250 iterations for each of the four structural combinations, see section DDCM parameterization). In each iteration, we applied a pixel-wise uniform jitter to each input parameter P, with the magnitude of the jittering varying from $\pm10$% (low variability), through $\pm20$% (medium variability) to $\pm30$% (high variability) provided by the sensitivity analysis performed earlier. At the pixel level (18 km), we first jittered the growth coefficients $b$ and $c$, retrieving the corresponding ${AGC}_{pot}$via the Chapman-Richards recovery curves to ensure physically consistent projections. Similarly, for linear trends in severity, return period, or net forest area, the slope and intercept were jittered by the same scalar value (different for each pixel) to avoid non-physical projections. The 95% confidence intervals for the European carbon sink were derived from the 2.5th and 97.5th percentiles of the resulting simulation ensemble.

**Recent increases in natural disturbances and harvests**. To produce Fig. 4c (blue axis on the left), we first extracted the ground-based data from [9] and then computed the total sum of the volume of wood lost due to natural disturbances from all countries in the EU-27 (column “Reported_GapFilled_m3” in their dataset) for each year from 2010 to 2019. The agent “other biotics” in their dataset has been discarded to match the convention of the Landsat-based map with the disturbance agents attributed (see previous section “partitioning between natural disturbances and harvests”). Then, for the Landsat-based estimation of the increase in natural disturbances (red axis on the right in Fig. 4c), we first discarded low-confidence pixels at 18 km (see section “Partitioning between natural disturbances and harvests”). Then, we spatially summed for each year (from 2010 to 2019) the severities associated with natural disturbances (storms and fire) in the EU-27. We divided by the forest area of EU-27 (we discarded the forest area of low-confidence pixels). We similarly produced Fig. 4d, except using the severities associated with harvests from 2010 to 2020 for the Landsat-based data (red axis on the right) and the roundwood removals from 2010 to 2020 extracted from Eurostat [13] for the ground-based data (blue axis on the left).

**Forest growth change.** We estimated forest growth change in Europe (Fig. 4a) with CCI-ESA data between 2016 (period 2015-2017) and 2020 (period 2019-2021) for five different age class: [5-10], [10-15], …, [25-30] years old. These forests have not been disturbed since (according to the disturbance map). Therefore, the AGC associated with their age represents their net forest growth. For each year (from 2015 to 2021), we first aggregated from 30 m to 18 km the mean AGC of forests severely disturbed since [5-10], [10-15], …, [25-30] years (therefore five maps per year). Then we calculated for each age class and each year the spatial mean of all 18 km pixels presents in a given biogeographical region. Fig. 4a shows the comparison for each age class between two periods: 2016 (mean of 2015-2017) and 2020 (mean of 2019-2021). We did not detect any significant change in net forest growth between these two periods ($\pm5\%$). This means for example that a 20 years old forest is associated, on average, with the same AGC whereas it lived in 2016 or in 2020.

**Scenarios 2025-2030.** We present five scenarios from 2025 to 2030 (Fig. 5). The business-as-usual (BaU) scenario assumes that the long-term trends in severity (including harvests & natural disturbances) continue unaffected. The collapse scenario assumes that the forest growth is 10% lower than BaU and that the trends in severity due to natural disturbances are 25% higher than BaU. The next two scenarios assume that the three billion trees pledge is respected (additional 15 MtCO_2_eq/year of carbon sink [14]) and we estimated the required reduction in harvest rates for each to reach the 2030 target in the EU-27 (detailed in Table 1). Finally, the last scenario (No-Harvest) corresponds to a 100% decrease of harvest from 2025 to 2030. For all scenarios, the trends in net forest area continue unaffected as they represent only a minor change in 2030 (see Table 1). To calculate the estimated decrease in harvest to reach the 2030 forest carbon sink target for the BaU and Collapse scenario, we assumed that the ratio $R$ at 18 km between natural disturbances and harvests is constant (supported by Fig. S13). For a given 18 km pixel, let $s\left( y \right)=Ay+B$ be the annual percentage of AGC loss (including harvests and natural disturbances) and $s_{red}\left( y \right)= A_{red}y+B_{red}$ be the annual percentage of AGC loss (including harvests and natural disturbances) with a reduction in harvest for $y>2025$. We define $w$ as the percentage of reduction in harvest from 2025 to 2030. We have:

$$\left\{ \begin{aligned} A_{red}= \frac{1}{1+R}(RA-\frac{w}{500}(2025A+B)) \\ B_{red}=B+\frac{2025}{1+R}(A+\frac{w}{500}(2025A+B)) \end{aligned} \right.$$

Because A, B and R are known, $w$ is estimated at 18 km with Monte-Carlo simulations so that the forest carbon sinks at 18 km projected in 2030 correspond to their baseline in 2016-2018. Values provided in Table 1 are the mean across biogeographical regions (or the EU-27) weighted by the local forest area at 18 km. The no-harvest scenario corresponds to $w=100\%$ all across Europe.

We demonstrate now the two relations. We first need to partition natural disturbances and harvests using their ratio R.

$$s\left( y \right)= \frac{R}{1+R}s\left( y \right)+\frac{1}{1+R}s(y)$$

The first member is the natural disturbances (they will not be modified), and the second is the harvests. Let’s introduce an intermediate variable $s_{red}^{har}\left( y \right)= A^{har}y+B^{har}$, with $s_{red}^{har}\left( y \right)$ the annual percentage of AGC loss only due to harvests in the scenario where we have a reduction in harvest for $y>2025$. Therefore:

$$s_{red}\left( y \right)= \frac{R}{1+R}s\left( y \right)+s_{red}^{har}\left( y \right)$$

The percentage of reduction in harvest from 2025 to 2030 is defined as:

$$w= \frac{s_{red}^{har}\left( 2025 \right)-s_{red}^{har}\left( 2030 \right)}{s_{red}^{har}\left( 2025 \right)}100$$

Using $s_{red}^{har}\left( 2025 \right)=\frac{1}{1+R}s(2025)$in the denominator only leads to the following:

$$A^{har}= -\frac{2025A+B}{500(1+R)}w$$

This is enough to find $A_{red}$ based on

$$s_{red}\left( y \right)=A_{red}y+B_{red}=\frac{R}{1+R}(Ay+B)+A^{har}y+B^{har}$$

Therefore $A_{red}=\frac{R}{1+R}A+A^{har}$ which corresponds to the first equation we proposed. Finally, $B_{red}=s_{red}\left( 2025 \right)-{2025A}_{red}= s\left( 2025 \right)-{2025A}_{red}$ based on the fact that $s_{red}\left( 2025 \right)=s\left( 2025 \right)$. This leads to the second equation.


**Forest carbon sources in 2030.** For each scenario (no-harvest, business-as-usual, collapse, and their respective reduced harvest versions – including the 3 billion trees), we calculated the percentage of forests acting as carbon sources as the total forest area of pixels associated with a negative forest carbon sink divided by the total forest area of Europe. This percentage has been calculated for each of the 1000 Monte-Carlo simulations to quantify the mean and the confidence intervals (2.5% and 97.5% percentile). The maps in Fig. 5 correspond to the mean stack of the 1000 Monte-Carlo simulations.

**REFERENCES**

[1] Avitabile, V., Pilli, R., Migliavacca, M. et al. Harmonised statistics and maps of forest biomass and increment in Europe. *Sci. Data* **11**, 274 (2024). <https://doi.org/10.1038/s41597-023-02868-8>
[2] FAO. Global Forest Resources Assessment 2020: Country Reports https://www.fao.org/forest-resources-assessment/fra-2020/country-reports/en/ (2020).
[3] Senf, C. and Seidl, R. Mapping the forest disturbance regimes of Europe. Nat. Sustain. 4, 63–70 (2021). https://doi.org/10.1038/s41893-020-00609-y
[4] Liu, S. et al. The overlooked contribution of trees outside forests to tree cover and woody biomass across Europe. Sci. Adv. 9, eadh4097 (2023). <https://doi.org/10.1126/sciadv.adh4097>
[5] Santoro, M.; Cartus, O. (2024): ESA Biomass Climate Change Initiative (Biomass_cci): Global datasets of forest above-ground biomass for the years 2010, 2015, 2016, 2017, 2018, 2019, 2020 and 2021, v5. NERC EDS Centre for Environmental Data Analysis, 22 August 2024. <https://dx.doi.org/10.5285/02e1b18071ad45a19b4d3e8adafa2817>
[6] <https://www.citepa.org/wp-content/uploads/publications/ccnucc/LULUCF_Background-d_ed2023_v2.zip>
[7] Lestang, T., et al. Computing return times or return periods with rare event algorithms. J. Stat. Mech. Theory Exp. 2018, 043213 (2018).
[8] Senf, C. and Seidl, R. (2021) Storm and fire disturbance in Europe: Distribution and trends. Global Change Biology. https://doi.org/10.1111/gcb.15679
[9] Patacca, M. et al. Significant increase in natural disturbance impacts on European forests since 1950. Glob. Chang. Biol. 29, 1359–1376 (2023). <https://doi.org/10.1111/gcb.16531>
[10] Verkerk, P. et al. Mapping wood production in European forests. For. Ecol. and Manage. 357, 228-238 (2015). <https://doi.org/10.1016/j.foreco.2015.08.007>
[11] Babst, F. et al. Twentieth century redistribution in climatic drivers of global tree growth. Sci. Adv. 5, eaat4313 (2019).
[12] Ritter, F. Technical note: A procedure to clean, decompose, and aggregate time series. Hydrol. Earth Syst. Sci. 27, 349–361 (2023).
[13] Eurostat, Roundwood removals by type of wood and assortment. https://doi.org/10.2908/FOR_REMOV. Last data access: 25/10/2024.
[14] Grassi, G., et al., Brief on the role of the forest-based bioeconomy in mitigating climate change through carbon storage and material substitution. Sanchez Lopez, J., Jasinevičius, G. and Avraamides, M. editor(s), European Commission, JRC124374 (2021). https://publications.jrc.ec.europa.eu/repository/handle/JRC124374

**Supplementary material:**

In the following, we provide supplementary material with a more technical focus.

**Flowchart & example of the DDCM procedure.** Fig. S1 & Fig. S2 present the DDCM procedure with a summary of the workflow and a detailed example of the estimation of the forest carbon sink of an 18 km forested pixel.

**Country attribution to historical trends in the forest sink.** Tables S1 to S4 give details of the UNFCCC data associated with each country based on their forest sink trend from 2010 to 2021 (used to produce Fig. 1c). Countries are separated into four groups: small decrease (Table S1), no trend (Table S2), large decrease (Table S3) and increase (Table S4) in the forest sink.

**Harmonization with NFI data.** Fig. S3 explains how the AGC maps from PlanetScope & CCI-ESA have been bias-corrected with NFI data, and how a forest mask at 30 m consistent with NFI data has been produced.

**UNFCCC reports at a regional scale.** Fig. S4 explains how the net change in the carbon stocks of living biomass has been partitioned into net change in the carbon stocks of above-ground and below-ground biomass, and how UNFCCC reports have been aggregated from the national to the regional scale. It shows the linear relationships between the forest sink components and ΔAGC, with a summary of the linear regression detailed in Table S5.

**Parameterization of the DDCM**. Fig. S5 shows the parameterization of the DDCM for different AGC maps (PlanetScope, CCI-ESA) at different scales (national, regional).

**Recovery curves coefficients.** Fig. S6 shows the values of the recovery curves coefficients for all different parameterizations of the DDCM.

**Forest sink target for 2030.** Fig. S7 explains how the forest sink target for 2030 has been calculated with respect to the reference years of 2016-2017-2018 used by the European Commission to compute the LULUCF target.

**Inconsistencies, missing data & UNFCCC corrections.** Fig. S8 shows the inconsistencies in the UNFCCC reports of France & Portugal, as well as in the AGC maps of CCI-ESA between 2010 and the period 2015-2021. It quantifies the percentage of missing values across Europe, and shows the anomalies with in-situ data in the Mediterranean biogeographical region (discarded from Fig. 2b due to a small sample size).

**Linear trend in the return interval of disturbances aggregated at 18 km.** Fig. S9 shows the trends in the return interval of disturbances aggregated at 18 km.

**Uncertainties in the in-situ age-AGC measurements**. Fig. S10 shows a sensitivity analysis performed on the in-situ dataset of age-AGC across Europe to estimate that the measurement uncertainty is between 20 and 30 MgC per sample depending on the age.

**Hungary case-study**. Fig. S11 shows the mean growth curve and mean percentage of AGC loss of Hungary compared to each of its neighboring countries. Hungary is the only country with a large forest area that reported an increase in the forest sink from 2010 to 2021 (UNFCCC, see Table S4 and Fig. S21). The reported forest area has only increased by 0.005% per year from 2010 to 2021. We currently do not explain the reported increase in the forest sink.

**Comparison between a Landsat-based harvest map and an NFI-based wood production map.** Fig. S12 shows a comparison between an NFI-based wood production map of Europe and a landsat-based harvest map. We flaged 18 km pixels that show a low agreement between the two maps (14% of Europe). These low-confidence pixels have been discarded from the study when partitioning natural and anthropogenic disturbances.

**Ratio between natural disturbances and harvests at 18 km**. Fig. S13 shows the 18 km map of the mean ratio between natural disturbances and harvests from 1986 to 2020. Low-confidence pixels flagged in Fig. S12 were discarded and imputed based on the ratio of their neighbors (which are high confidence pixels) sharing similar wood production.

**Changes in the ratio between natural disturbances and harvests from 1986 to 2020.** Fig. S14 shows the annual ratio between natural disturbances and harvests from 1986 to 2020 across Europe (low-confidence pixels were discarded). The mean value over the period (ratio of 17%) remarkably matches the ground-based value (16 %).

**Sensitivy test of the DDCM to different starting assumptions.** Fig. S15 shows results from the DDCM with two different starting assumptions: either (i) The trends in AGC loss are derived at 54 km instead of 18 km (higher sample size, lower spatial heterogeneity), or (ii) The trends in AGC loss have been upscaled additively instead of multiplicatively ($s\left( y \right)=f_{loss}\left( y \right)+\beta instead of s\left( y \right)=\beta f_{loss}(y)$, with $f_{loss}(y)$ the linear trend at 18 km in the percentage of forest cover loss). Both alternate models show similar results compared to the main study, however their goodness of fit with the country reports to UNFCCC are worse. We therefore kept the original approach only.

**Proof of concept for the DDCM Model.** Fig. S16 demonstrates that the DDCM model successfully captures the decline in the forest carbon sink while being parametrizated with the UNFCCC data for the year 2010 only.

**Inconsistencies in biomass change detection using remote sensing.** Fig. S17 shows the inaccuracies of CCI-ESA maps (v5) in capturing AGC change in Europe, and proves that the DDCM projections accurately capture them.

**Propagation of uncertainties.** Fig. S18 shows the sensitivity analysis performed to estimate the natural uncertainty of each input parameter. Fig. S19 shows the magnitude of jittering of each input paramer derived from Fig. S18 to perform the 1000 Monte-Carlo simulations.

**DDCM outputs for each country.** Fig. S20 to Fig. S33 show the individual forest sink components for each country, with the comparison with UNFCCC reports across the historical period (when available).

**Fig. S1. Flowchart of the DDCM procedure.** This flowchart explains the DDCM procedure from raw data to forest sink estimates. Computation of the ratio between natural disturbances and harvests has not be shown for clarity (Fig. S12-13), as well at the sensitivity analysis (Fig. S15) or data inconsistencies (Fig. S8). The variable **s** refers to the percentage of AGC loss, and $\boldsymbol{\tau}$ to the return interval of the disturbances (for a 30-year window), both defined at 18 km. The correction factors (α,β) are defined at a country or a biogeographical region scale (see methods).

**Fig. S2. Detailed example of the DDCM procedure for a given forested pixel at 18 km.** Use of the flowchart presented in Fig. S1 on a forested pixel at 18 km. The parametrization is a bootstrap conducted on the scale of a country or a biogeographical region to adjust the correction factors ($\alpha$,$\beta$) so that the difference between simulated and observed (UNFCCC) annual changes in AGC is minimized across the historical period (2010-2021). Each forest sink component (ΔHWP, ΔSoil, ΔBGC and ΔDWL in panel **B**) is computed based on ΔAGC with the linear relationships detailed in Fig. S4d-g and Table S5.

|  |  |  |  |  |  |  |  |  |  |
| --- | --- | --- | --- | --- | --- | --- | --- | --- | --- |

| Countries with a small decrease in their forest carbon sink (from -5 to 0%/yr) | | | | | | |
| --- | --- | --- | --- | --- | --- | --- |
| year | Belarus | United-kingdom | Croatia | Ireland | Lithuania | Netherlands |
| **Forest Sink (MtCO2eq)** |  |  |  |  |  |  |
| 2010 | 60.34 | 21.65 | 7.98 | 4.24 | 11.33 | 2.29 |
| 2011 | 59.52 | 21.71 | 6.99 | 4.36 | 12.04 | 2.26 |
| 2012 | 58.02 | 21.41 | 6.90 | 4.48 | 11.48 | 2.25 |
| 2013 | 60.20 | 21.30 | 7.02 | 4.80 | 11.49 | 2.29 |
| 2014 | 59.76 | 21.14 | 6.84 | 4.58 | 10.77 | 2.25 |
| 2015 | 59.12 | 21.11 | 6.71 | 5.15 | 9.81 | 2.17 |
| 2016 | 54.04 | 21.05 | 6.58 | 5.26 | 8.71 | 2.18 |
| 2017 | 50.09 | 20.90 | 6.61 | 4.19 | 8.51 | 2.15 |
| 2018 | 53.62 | 20.77 | 6.26 | 3.71 | 8.29 | 2.12 |
| 2019 | 49.70 | 20.66 | 6.52 | 3.19 | 7.87 | 2.12 |
| 2020 | 51.92 | 20.31 | 6.85 | 2.91 | 8.54 | 2.12 |
| 2021 | 52.74 | 20.13 | 6.57 | 2.16 | 8.10 | 1.94 |
| **slope (%/yr)** | **-1.59** | **-0.61** | **-1.15** | **-3.55** | **-3.39** | **-1.08** |
| ***p-value*** | ***< 0.001*** | ***< 0.001*** | ***0.010*** | ***0.012*** | ***< 0.001*** | ***< 0.001*** |
| **Forest Area (kha)** |  |  |  |  |  |  |
| 2010 | 8915.29 | 3538.36 | 2361.57 | 731.58 | 2153.99 | 374.78 |
| 2011 | 8979.79 | 3552.75 | 2367.42 | 736.63 | 2161.97 | 375.35 |
| 2012 | 9083.29 | 3566.48 | 2372.20 | 742.48 | 2173.55 | 375.91 |
| 2013 | 9101.49 | 3578.59 | 2379.18 | 747.13 | 2179.54 | 373.37 |
| 2014 | 9281.09 | 3587.42 | 2387.48 | 752.89 | 2187.53 | 370.82 |
| 2015 | 9402.20 | 3590.03 | 2393.62 | 758.38 | 2196.72 | 368.27 |
| 2016 | 9492.50 | 3592.80 | 2395.01 | 764.08 | 2201.91 | 365.73 |
| 2017 | 9537.30 | 3598.25 | 2398.94 | 769.39 | 2209.89 | 365.24 |
| 2018 | 9575.20 | 3606.42 | 2400.66 | 773.23 | 2210.69 | 364.76 |
| 2019 | 9668.40 | 3614.87 | 2401.16 | 776.65 | 2214.29 | 364.28 |
| 2020 | 9827.60 | 3625.15 | 2401.49 | 778.99 | 2221.47 | 363.80 |
| 2021 | 9933.40 | 3635.75 | 2401.87 | 781.00 | 2230.66 | 363.31 |
| **slope (%/yr)** | **1.03** | **0.22** | **0.16** | **0.64** | **0.31** | **-0.35** |
| ***p-value*** | ***< 0.001*** | ***< 0.001*** | ***< 0.001*** | ***< 0.001*** | ***< 0.001*** | ***< 0.001*** |

**Table S1. Countries with a small decrease in their forest sink from 2010 to 2021 (UNFCCC data).** UNFCCC data used in Fig. 1d to attribute a small decrease in the forest sink of 9 countries in Europe (linear regression, p-value < 0.05, slope between -5 and 0 % / yr)

| Countries with a small decrease in their forest carbon sink (from -5 to 0%/yr) (cont.) | | | |
| --- | --- | --- | --- |
| year | Norway | Poland | Sweden |
| **Forest Sink (MtCO2eq)** |  |  |  |
| 2010 | 29.18 | 38.23 | 57.40 |
| 2011 | 30.31 | 43.59 | 57.51 |
| 2012 | 26.91 | 44.05 | 58.00 |
| 2013 | 27.01 | 46.43 | 55.99 |
| 2014 | 23.43 | 39.82 | 54.64 |
| 2015 | 19.13 | 35.89 | 53.89 |
| 2016 | 18.79 | 47.05 | 52.10 |
| 2017 | 17.72 | 44.90 | 45.30 |
| 2018 | 18.74 | 43.73 | 43.68 |
| 2019 | 20.64 | 25.48 | 44.60 |
| 2020 | 24.05 | 26.12 | 47.07 |
| 2021 | 20.63 | 27.10 | 46.59 |
| **slope (%/yr)** | **-3.17** | **-3.01** | **-2.36** |
| ***p-value*** | ***0.008*** | ***0.032*** | ***< 0.001*** |
| **Forest Area (kha)** |  |  |  |
| 2010 | 12139.08 | 9304.76 | 28081.10 |
| 2011 | 12139.37 | 9329.17 | 28093.58 |
| 2012 | 12139.89 | 9353.73 | 28096.90 |
| 2013 | 12140.47 | 9369.40 | 28108.70 |
| 2014 | 12140.32 | 9382.58 | 28107.18 |
| 2015 | 12139.30 | 9395.17 | 28116.19 |
| 2016 | 12136.81 | 9381.98 | 28119.82 |
| 2017 | 12134.79 | 9425.73 | 28127.73 |
| 2018 | 12132.74 | 9434.08 | 28141.87 |
| 2019 | 12131.06 | 9439.12 | 28156.73 |
| 2020 | 12129.82 | 9442.87 | 28156.55 |
| 2021 | 12129.30 | 9450.11 | 28170.58 |
| **slope (%/yr)** | **-0.01** | **0.14** | **0.03** |
| ***p-value*** | ***< 0.001*** | ***< 0.001*** | ***< 0.001*** |

**Table S1 (Cont.). Countries with a small decrease in their forest sink from 2010 to 2021 (UNFCCC data).** UNFCCC data used in Fig. 1d to attribute a small decrease in the forest sink of 9 countries in Europe (linear regression, p-value < 0.05, slope between -5 and 0 % / yr)

| G1: Countries with no trend in their forest carbon sink (p > 0.05) | | | | | | |
| --- | --- | --- | --- | --- | --- | --- |
| year | Belgium | Bulgaria | Switzerland | Germany | Denmark | Spain |
| **Forest Sink (MtCO2eq)** |  |  |  |  |  |  |
| 2010 | 2.04 | 11.36 | 3.95 | 52.67 | 2.32 | 43.89 |
| 2011 | 2.03 | 8.08 | 2.53 | 56.43 | 3.33 | 43.28 |
| 2012 | 2.02 | 8.01 | 3.64 | 63.70 | 3.69 | 42.60 |
| 2013 | 2.11 | 7.89 | 3.40 | 67.07 | 3.51 | 42.15 |
| 2014 | 2.09 | 7.93 | 2.06 | 59.37 | 4.13 | 42.53 |
| 2015 | 2.04 | 8.07 | 3.62 | 60.83 | 4.20 | 43.04 |
| 2016 | 2.25 | 9.58 | 3.48 | 62.90 | 3.32 | 42.73 |
| 2017 | 2.14 | 9.77 | 3.38 | 59.46 | 2.76 | 43.42 |
| 2018 | 2.12 | 9.62 | 2.17 | 57.04 | 2.20 | 43.43 |
| 2019 | 2.00 | 9.37 | 3.15 | 55.55 | 2.60 | 42.67 |
| 2020 | 1.87 | 9.52 | 2.84 | 47.87 | 2.32 | 41.69 |
| 2021 | 1.88 | 9.56 | 3.00 | 50.49 | 3.02 | 41.57 |
| **slope (%/yr)** | **-0.49** | **0.73** | **-1.41** | **-1.17** | **-2.05** | **-0.25** |
| ***p-value*** | ***0.270*** | ***0.502*** | ***0.371*** | ***0.130*** | ***0.232*** | ***0.067*** |
| **Forest Area (kha)** |  |  |  |  |  |  |
| 2010 | 711.69 | 3892.55 | 1246.14 | 10939.93 | 627.98 | 16683.66 |
| 2011 | 710.42 | 3895.21 | 1248.08 | 10942.50 | 631.33 | 16702.81 |
| 2012 | 709.16 | 3897.87 | 1250.03 | 10945.03 | 632.79 | 16721.96 |
| 2013 | 709.62 | 3900.52 | 1251.96 | 10947.54 | 637.51 | 16746.14 |
| 2014 | 710.07 | 3903.18 | 1253.85 | 10950.05 | 637.55 | 16770.32 |
| 2015 | 710.53 | 3905.84 | 1255.78 | 10952.55 | 637.79 | 16794.49 |
| 2016 | 709.96 | 3908.49 | 1257.72 | 10960.91 | 637.73 | 16860.65 |
| 2017 | 709.39 | 3911.15 | 1259.66 | 10969.23 | 638.85 | 16926.81 |
| 2018 | 708.83 | 3913.81 | 1261.65 | 10977.55 | 639.36 | 16992.96 |
| 2019 | 708.29 | 3916.46 | 1263.66 | 10985.85 | 640.36 | 17059.12 |
| 2020 | 707.75 | 3919.12 | 1265.68 | 10994.15 | 641.58 | 17125.27 |
| 2021 | 707.22 | 3920.20 | 1267.70 | 11003.17 | 646.24 | 17191.43 |
| **slope (%/yr)** | **-0.04** | **0.07** | **0.16** | **0.05** | **0.20** | **0.28** |
| ***p-value*** | ***< 0.001*** | ***< 0.001*** | ***< 0.001*** | ***< 0.001*** | ***< 0.001*** | ***< 0.001*** |

**Table S2. Countries without significant trend in their forest sink from 2010 to 2021 (UNFCCC data).** UNFCCC data used in Fig. 1d to attribute an absence of trend in the forest sink of 15 countries in Europe (linear regression, p-value > 0.05).

| Countries with no trend in their forest carbon sink (p > 0.05) (cont.) | | | | | | |
| --- | --- | --- | --- | --- | --- | --- |
| year | Greece | Italy | Luxembourg | Latvia | Portugal | Romania |
| **Forest Sink (MtCO2eq)** |  |  |  |  |  |  |
| 2010 | 2.56 | 36.63 | 0.26 | 6.91 | 8.65 | 34.70 |
| 2011 | 2.51 | 33.12 | 0.36 | 7.55 | 3.51 | 33.30 |
| 2012 | 2.14 | 28.50 | 0.43 | 7.98 | 3.65 | 34.42 |
| 2013 | 2.13 | 38.40 | 0.59 | 7.25 | 2.72 | 34.77 |
| 2014 | 2.14 | 39.39 | 0.50 | 3.03 | 3.38 | 35.81 |
| 2015 | 2.12 | 40.30 | 0.45 | 4.66 | 2.99 | 35.40 |
| 2016 | 2.14 | 39.38 | 0.52 | 6.17 | 1.31 | 36.35 |
| 2017 | 2.36 | 25.22 | 0.43 | 8.31 | -0.69 | 34.90 |
| 2018 | 2.45 | 41.49 | 0.29 | 7.49 | 1.03 | 32.94 |
| 2019 | 2.56 | 38.94 | 0.41 | 8.41 | 1.55 | 33.65 |
| 2020 | 2.44 | 32.26 | 0.50 | 5.28 | 1.63 | 32.90 |
| 2021 | 2.58 | 30.41 | 0.65 | 3.82 | 5.11 | 32.46 |
| **slope (%/yr)** | **0.76** | **-0.30** | **3.60** | **-1.63** | **-6.93** | **-0.44** |
| ***p-value*** | ***0.315*** | ***0.819*** | ***0.167*** | ***0.474*** | ***0.101*** | ***0.141*** |
| **Forest Area (kha)** |  |  |  |  |  |  |
| 2010 | 3440.64 | 8986.39 | 93.33 | 3238.43 | 4336.90 | 6969.40 |
| 2011 | 3444.51 | 9031.83 | 93.33 | 3242.80 | 4338.75 | 6974.41 |
| 2012 | 3448.39 | 9077.27 | 93.32 | 3247.17 | 4340.59 | 6979.42 |
| 2013 | 3452.27 | 9122.71 | 93.28 | 3251.54 | 4341.58 | 6980.68 |
| 2014 | 3456.15 | 9168.15 | 93.24 | 3250.22 | 4342.56 | 6981.93 |
| 2015 | 3460.03 | 9213.58 | 93.20 | 3248.89 | 4343.55 | 6983.19 |
| 2016 | 3463.91 | 9259.02 | 93.18 | 3247.57 | 4343.00 | 6984.44 |
| 2017 | 3467.79 | 9304.46 | 93.16 | 3246.25 | 4342.46 | 6985.70 |
| 2018 | 3471.60 | 9349.90 | 93.15 | 3244.92 | 4341.92 | 6986.96 |
| 2019 | 3475.57 | 9395.34 | 93.13 | 3253.51 | 4341.37 | 6988.21 |
| 2020 | 3479.35 | 9440.78 | 93.11 | 3262.09 | 4340.83 | 6989.47 |
| 2021 | 3484.26 | 9486.22 | 93.09 | 3270.68 | 4340.29 | 6990.72 |
| **slope (%/yr)** | **0.11** | **0.51** | **-0.03** | **0.06** | **0.01** | **0.02** |
| ***p-value*** | ***< 0.001*** | ***< 0.001*** | ***< 0.001*** | ***0.004*** | ***0.175*** | ***< 0.001*** |

**Table S2 (cont.). Countries without significant trend in their forest sink from 2010 to 2021 (UNFCCC data).** UNFCCC data used in Fig. 1d to attribute an absence of trend in the forest sink of 15 countries in Europe (linear regression, p-value > 0.05).

| G1: Countries with no trend in their forest carbon sink (p > 0.05) (cont.) | | | |
| --- | --- | --- | --- |
| year | Slovakia | Slovenia | Ukraine |
| **Forest Sink (MtCO2eq)** |  |  |  |
| 2010 | 4.23 | 7.27 | 30.56 |
| 2011 | 4.55 | 7.21 | 28.57 |
| 2012 | 6.09 | 7.20 | 27.80 |
| 2013 | 6.55 | 5.38 | 26.54 |
| 2014 | 4.38 | -0.64 | 27.63 |
| 2015 | 4.98 | -0.74 | 24.07 |
| 2016 | 4.83 | -0.88 | 23.42 |
| 2017 | 4.76 | -1.01 | 27.29 |
| 2018 | 3.85 | -1.09 | 28.25 |
| 2019 | 4.61 | 3.32 | 29.07 |
| 2020 | 6.88 | 3.15 | 34.23 |
| 2021 | 6.68 | 3.14 | 36.18 |
| **slope (%/yr)** | **1.87** | **-9.19** | **1.72** |
| ***p-value*** | ***0.342*** | ***0.085*** | ***0.151*** |
| **Forest Area (kha)** |  |  |  |
| 2010 | 2011.25 | 1206.55 | 10601.10 |
| 2011 | 2012.34 | 1206.50 | 10611.30 |
| 2012 | 2014.06 | 1206.45 | 10621.40 |
| 2013 | 2015.37 | 1206.85 | 10624.17 |
| 2014 | 2017.11 | 1207.25 | 10630.28 |
| 2015 | 2020.12 | 1207.65 | 10630.43 |
| 2016 | 2022.52 | 1208.05 | 10690.40 |
| 2017 | 2024.37 | 1207.03 | 10710.99 |
| 2018 | 2026.03 | 1206.00 | 10685.56 |
| 2019 | 2027.10 | 1204.97 | 10686.79 |
| 2020 | 2027.85 | 1204.65 | 10689.33 |
| 2021 | 2028.51 | 1204.33 | 10692.54 |
| **slope (%/yr)** | **0.09** | **-0.02** | **0.09** |
| ***p-value*** | ***< 0.001*** | ***0.039*** | ***< 0.001*** |

**Table S2 (cont.). Countries without significant trend in their forest sink from 2010 to 2021 (UNFCCC data).** UNFCCC data used in Fig. 1d to attribute an absence of trend in the forest sink of 15 countries in Europe (linear regression, p-value > 0.05).

| Countries with a large decrease in their forest carbon sink (< -5%/yr) | | | | | |
| --- | --- | --- | --- | --- | --- |
| year | Austria | Czechia | Estonia | Finland | France |
| **Forest Sink (MtCO2eq)** |  |  |  |  |  |
| 2010 | 21.54 | 7.28 | 7.28 | 41.18 | 65.22 |
| 2011 | 17.10 | 7.74 | 6.86 | 40.24 | 65.78 |
| 2012 | 7.51 | 7.80 | 5.09 | 41.85 | 66.29 |
| 2013 | 7.99 | 7.14 | 4.76 | 34.84 | 74.10 |
| 2014 | 9.35 | 7.10 | 2.32 | 35.11 | 62.70 |
| 2015 | 8.31 | 6.89 | 3.04 | 30.77 | 58.58 |
| 2016 | 8.78 | 6.03 | 2.31 | 27.80 | 48.78 |
| 2017 | 5.06 | 4.45 | 2.18 | 25.15 | 38.09 |
| 2018 | -3.09 | -1.14 | 0.48 | 16.35 | 32.83 |
| 2019 | -0.29 | -7.91 | 1.39 | 21.62 | 30.45 |
| 2020 | 7.07 | -11.33 | -0.11 | 24.21 | 33.41 |
| 2021 | 12.26 | -8.47 | -0.13 | 14.41 | 29.58 |
| **slope (%/yr)** | **-7.58** | **-14.91** | **-10.12** | **-5.73** | **-5.78** |
| ***p-value*** | ***0.040*** | ***< 0.001*** | ***< 0.001*** | ***< 0.001*** | ***< 0.001*** |
| **Forest Area (kha)** |  |  |  |  |  |
| 2010 | 3994.47 | 2657.38 | 2442.88 | 21943.12 | 18214.85 |
| 2011 | 3996.75 | 2659.84 | 2444.03 | 21927.98 | 18254.09 |
| 2012 | 3999.03 | 2661.89 | 2445.26 | 21915.03 | 18294.12 |
| 2013 | 4001.31 | 2663.73 | 2446.30 | 21903.57 | 18317.08 |
| 2014 | 4003.59 | 2666.38 | 2447.02 | 21893.56 | 18350.02 |
| 2015 | 4005.88 | 2668.39 | 2447.38 | 21885.48 | 18384.71 |
| 2016 | 4008.16 | 2669.85 | 2447.33 | 21878.11 | 18419.51 |
| 2017 | 4010.44 | 2671.66 | 2446.97 | 21870.97 | 18454.73 |
| 2018 | 4012.72 | 2673.39 | 2446.82 | 21863.84 | 18492.12 |
| 2019 | 4015.00 | 2675.67 | 2446.91 | 21857.80 | 18529.48 |
| 2020 | 4017.28 | 2677.33 | 2447.06 | 21851.60 | 18566.87 |
| 2021 | 4019.56 | 2678.80 | 2447.41 | 21845.79 | 18604.35 |
| **slope (%/yr)** | **0.06** | **0.07** | **0.01** | **-0.04** | **0.19** |
| ***p-value*** | ***< 0.001*** | ***< 0.001*** | ***0.003*** | ***< 0.001*** | ***< 0.001*** |

**Table S3. Countries with a large decrease in their forest sink from 2010 to 2021 (UNFCCC data).** UNFCCC data used in Fig. 1d to attribute a large decrease in the forest sink of 5 countries in Europe (linear regression, p-value < 0.05, slope below -5 %/yr)

| Countries with an increase in their forest carbon sink (> 0%/yr) | | |
| --- | --- | --- |
| year | Hungary | Liechtenstein |
| **Forest Sink (MtCO2eq)** |  |  |
| 2010 | 4.30 | -0.0074 |
| 2011 | 3.81 | -0.0111 |
| 2012 | 4.57 | -0.0113 |
| 2013 | 3.75 | -0.0038 |
| 2014 | 4.94 | -0.0037 |
| 2015 | 5.66 | 0.0016 |
| 2016 | 4.73 | 0.0034 |
| 2017 | 5.55 | 0.0018 |
| 2018 | 5.02 | -0.0092 |
| 2019 | 5.65 | 0.0004 |
| 2020 | 7.34 | 0.0076 |
| 2021 | 7.60 | 0.0118 |
| **slope (%/yr)** | **7.95** | **15.27** |
| ***p-value*** | ***< 0.001*** | ***0.003*** |
| **Forest Area (kha)** |  |  |
| 2010 | 2170.46 | 6.15 |
| 2011 | 2173.18 | 6.17 |
| 2012 | 2176.83 | 6.18 |
| 2013 | 2183.51 | 6.19 |
| 2014 | 2183.56 | 6.21 |
| 2015 | 2184.09 | 6.22 |
| 2016 | 2180.37 | 6.23 |
| 2017 | 2177.00 | 6.24 |
| 2018 | 2172.32 | 6.25 |
| 2019 | 2171.61 | 6.27 |
| 2020 | 2175.74 | 6.28 |
| 2021 | 2181.97 | 6.28 |
| **slope (%/yr)** | **0.01** | **0.20** |
| ***p-value*** | ***0.794*** | ***< 0.001*** |

**Table. S4. Countries with an increase in their forest sink from 2010 to 2021 (UNFCCC data).** UNFCCC data used in Fig. 1d to attribute an increase in the forest sink of Hungary and Liechtenstein in Europe (linear regression, p-value < 0.05, slope above 0 %/yr).

**Fig. S3. Harmonization of the 30 m forest mask and the AGC maps from CCI-ESA & PlanetScope with NFI data. A,** Above-ground biomass (AGC, in MgC/haF, Megagram Carbon per hectare of forests) for the reference year 2020 from the harmonized NFI dataset on a sub-national scale (288 departmental units distributed among 40 countries). **B**, Correction factor for the AGC from CCI-ESA maps. **C**, Correction factor for the AGC from the PlanetScope map. For each unit, the correction is $\frac{{AGB}_{NFI}}{{AGB}_{RS}}$ with ${AGB}_{RS}$ the raw AGC from CCI-ESA (year 2020) or PlanetScope (year 2019), and ${AGB}_{NFI}$ the AGC from the NFI dataset. **D**, Scatter plot of the corrected AGC maps (aggregation performed per unit) versus the AGC from the NFI dataset. **E**, Forest cover for the reference year 2020 from the NFI dataset. **F**, Threshold above which the mean of the forest mask from Senf & Seidl (2021) & Liu et al. (2023) is set to 1 (otherwise 0). This threshold is calculated for each unit so that the aggregated sum of the forest mask at 30 m corresponds to the forest area reported by NFI. **G**, Scatter plot of the forest cover from the harmonized forest mask at 30 m (aggregation performed per unit) versus the forest cover from the NFI dataset

**Fig. S4. UNFCCC reports, computation of the BGC & AGC components, and linear relationships between forest sink components and annual changes in AGC. a,** Ratio of belowground biomass (BGC) to AGC calculated based on the FAO-FRA reports of 2020 (The year 2015 has been used instead for Serbia, Albania, Portugal and Greece as the year 2020 was not available yet. Blank areas correspond to no data available). This ratio is used to partition the annual carbon stock change in living biomass into the annual carbon stock change in AGC & BGC. **b**, Contribution of the forest area of each country to each bioregion, which allows to calculate each component of the forest sink (from the country reports to UNFCCC) for each biogeographical region. **c**, Linear relationships obtained from the UNFCCC reports associated with each biogeographical region of the forest sink FS (sum of annual carbon stock changes in AGC, BGC, Harvested Wood Products (HWP), Deadwood and Litter (DWL) and organic and mineral soil (soil)) versus annual carbon stock changes in AGC for 12 data points (years from 2010 to 2021). **d,e,f,g**, Similar relationships but for ΔHWP, ΔSoil, ΔBGC and ΔDWL versus ΔAGC, respectively. The mean is calculated instead if the relationship is non-significant (p > 0.05). The coefficients from the linear regression are presented in Table S5.

| $\boldsymbol{\Delta BGC vs \Delta AGC}$ | **Alpine** | **Continental** | **Mediterranean** | | **Boreal** | **Atlantic** |
| --- | --- | --- | --- | --- | --- | --- |
| slope | 0.2553 | 0.2215 | 0.2832 | 0.2759 | | 0.2726 |
| intercept  p-value  r^2^ | -0.0055 0.0026 0.9986 | -0.0128 < 0.0001 0.9988 | 0.0211 < 0.0001 0.9958 | -0.0024 0.0023 0.9995 | | 0.0106 < 0.0001 0.9980 |
|  |  |  |  |  | |  |
| $\boldsymbol{\Delta DWL vs \Delta AGC}$ |  |  |  |  | |  |
| slope | -0.0572 | -0.1855 | 0.0094 | 0 | | -0.0932 |
| intercept  p-value  r^2^ | 0.102 < 0.0001 0.5249 | 0.1792 < 0.0001 0.5688 | 0.0114 < 0.0001 0.6068 | 0.0445 0.2007 NA | | 0.1916 < 0.0001 0.4613 |
| $\boldsymbol{\Delta HWP vs \Delta AGC}$ |  |  |  |  | |  |
| slope | -0.0804 | -0.1810 | -0.1864 | -0.1027 | | 0.0123 |
| intercept  p-value  r^2^ | 0.0969 < 0.0001 0.5378 | 0.1952 < 0.0001 0.8332 | 0.1009 0.0012 0.5529 | 0.0954 < 0.0001 0.8377 | | 0.0638 < 0.0001 0.1151 |
| $\boldsymbol{\Delta Soil vs \Delta AGC}$ |  |  |  |  | |  |
| slope | 0 | 0 | 0 | 0.0993 | | -0.0545 |
| intercept  p-value  r^2^ | 0.0391 0.2293 NA | 0.0989 0.4160 NA | 0.0325 0.9846 NA | 0.0182 0.0436 0.6061 | | 0.0651 < 0.0001 0.6172 |

**Table S5. Linear regression summary.** Linear regression coefficients obtained for the relationships shown in Fig. S4D-G based on 12 annual points from the UNFCCC reports aggregated from national to regional scale (forest sink components in MgC/haF). If the trend is non-significant (p > 0.05), the intercept is replaced by the mean and the slope is set to 0 ($r^{2}$ has been discarded for clarity reasons).

**Fig. S5. Parameterization of the DDCM.** The parameterization of the DDCM aims to estimate ${AGC}_{pot}$ and the annual percentage of AGC loss ($s\left( y \right)$) based the following scalings: ${AGC}_{pot}= \alpha{AGC}_{75\%}$ and $s\left( y \right)=\beta f_{loss}(y)$. The variable ${AGC}_{75\%}$ is the 75% percentile of the AGC of all 30 m forests undisturbed since 1986 inside an 18 km pixel and $f_{loss}(y)$ is the linear trend at 18 km in the percentage of forest cover loss (based on six 30 years moving averages of Landsat data). The two corrections factors $\alpha$ and $\beta$ have been estimated with a bootstrap analysis so that the RMSE between simulated and observed (UNFCCC) ΔAGC is minimized across the historical period (2010-2021). The bootstrap has been conducted on the national (panels **A** & **B**) and regional (panels **C** & **D**) scales for two different AGC products, CCI-ESA (panels **B** & **D)** and PlanetScope (panels **A** & **C)**. The first row is associated with $\alpha$ values, the second row is associated with$\beta$ values and the third row with RMSE values.

**Fig. S6. Chapman-Richards coefficients from the recovery curves.** The Chapman-Richards growth function is decribed as $AGC\left( t \right)={AGC}_{pot}{(1-e^{-b})}^{c}$. **A**, Growth coefficients obtained from the parameterization of the DDCM at a country scale for the PlanetScope AGC map. **B**, Growth coefficients obtained from the parameterization of the DDCM at a country scale for the CCI-ESA AGC maps. **C**, growth coefficients obtained from the parameterization of the DDCM at a regional scale for the PlanetScope AGC map. **D**, growth coefficients obtained from the parameterization of the DDCM at a regional scale for the CCI-ESA AGC maps. Missing values in these maps (Fig. S8) will be filled with spatial interpolations before simulating the AGC from 2010 to 2030.

**Fig. S7. Estimation of the forest sink target by 2030. A,** The reference years 2016, 2017 & 2018 have been used by the European Commission to estimate the LULUCF sink target by 2030 (310 MtCO_2_eq/year) as shown with UNFCCC reports of the EU-27 countries. **B**, Forest sink target of 2030 for the EU-27 (392 MtCO_2_eq/year) estimated on the same reference years (2016, 2017 & 2018) but computed instead on the forest sink (sum of net annual carbon stock change in living biomass, deadwood, litter, soils (organic & mineral) and harvested wood products).

**Fig. S8. Inconsistencies & missing values in the datasets used in the study.** **A**, Anomalies between simulated and observed AGC (in-situ age-AGC dataset) for the Mediterranean region, discarded from Fig. 2C because of a small sample size. **B**, Mean values of AGC maps (bias-corrected with NFI data) across Europe (points). Lines represent AGC simulations from the DCCM with a parametrization conducted at different scales (national, regional) on different AGC maps (CCI-ESA, PlanetScope). Because these simulations are matching the historical trends reported in UNFCCC data from 2010 to 2021, this figure shows that the 2010 CCI-ESA map has a ~6% systematic positive bias across Europe. **C,** The CRF reports from UNFCCC give details of the forest sink in their Table 4a and details of the LULUCF sink in their Table 4. The net CO_2_ removals from “forest land” in their Table 4 must be rigourously the same than the net CO_2_ removals in their Table 4a (for the row “total forest land”), which is not the case here for Portugal (we divided by the forest area to allow comparison between countries). The LULUCF budget of Portugal has been therefore corrected based on the forest sink details of their Table 4a. **D**, Percentage of missing values (at 18 km) for the five different biogeographical regions and three parameters. These missing values have been filled out with spatial interpolations. **E** and **F**, France has reported a forest area including overseas territories to UNFCCC. We show this with an internal document of the CITEPA which reported the same forest sink than the UNFCCC report from France (panel **E**) but for the forest area of metropolitan France (panel **F**). We corrected the annual forest area of France using this document.

**Fig. S9. Linear trends in the return interval of aggregated disturbances at 18 km.** A time-series analysis has been conducted on the annual forest loss aggregated from 30 m to 18 km based on Landsat data (see Fig. S2 for a typical time-series). For a given 30-years window, the return interval is the time-series length (30 years) divided by the number of years associated with a forest cover loss above the mean loss. This figure shows the direction of the trends computed for six return intervals from 1986 to 2020 at 18 km. The lack of visible trends is due to two issues: (i) when disturbances are spatially aggregated (here 30 m to 18 km), the return interval of the disturbance partially loses its meaning, and (ii) 30 years are a short period to assess changes in disturbance return intervals. Some extreme events at 30 m have a typical return period of a century.

**Fig. S10. Analysis of the measurement uncertainties in the age-AGC in-situ dataset.** Uncertainties were not provided in the age-AGC in-situ datasets, we therefore conducted an analysis to estimate it. For each area of 100 km^2^ in Europe, we selected tree samples sharing similar age within $\pm2$ years. Because these trees share similar age at similar locations, we tested the variability in their AGC. To achieve this, we subtracted the mean AGC from each set to produce anomalies. The panels **A-J** show the stacked anomalies from all available sets, with an age varying from $10\pm2$ years to 100$\pm2$ years. Panel **K** shows the Mean Absolute Deviation for the stacked sets for each age bin. The uncertainty range is therefore estimated between 20 and 30 MgC for each in-situ sample (depending on the age).

**Fig. S11. Case-study: Hungary. A,** Mean growth curve of Hungary (in red) compared to its neighbors (Slovakia, Austria, Romania, Croatia, Slovenia, Ukraine and Serbia). **B,** Mean percentage of AGC loss of Hungary compared to its neighbors. The average is performed across all parameterizations (PlanetScope & CCI-ESA datasets, national & regional scales). Because Hungary shares similar growth rates compared to its neighbors, has slightly higher disturbance rates, and a limited forest area expansion (Fig. 1D), we do not explain the reported increase in the forest sink of Hungary from 2010 to 2021 (UNFCCC, see Fig. S21).

**Fig. S12. Comparison of a Landsat-based (Seidl et al., 2024) estimation of forest cover loss due to harvest with an NFI-based (Verkerk et al., 2015) estimation of wood production.** We first computed the sum of each product across the same period (2000-2010) and same resolution (18 km). Then we produced a quantile map of each to be able to compare them. We show here that 14% of pixels are associated with a low confidence (disagreement of more than 0.4 in the Landsat-based attribution of harvests). We discarded these pixels when producing Fig. 1C, Fig. 3B-C and Fig. S13.

**Fig. S13. Creation of a constant ratio between natural disturbances and harvests at 18 km.** Using the Landsat-based map of attribution of disturbances to speficic agents (Seidl et al., 2024), severities that are associated with natural disturbances (storms & fires) have been summed in each 18 km pixel, then divided by the sum of severities associated with harvests. We discarded low-confidence pixels (see Fig. S12), and replaced their ratio with the median ratio of pixels sharing similar wood production (Verkerk et al., 2015) but flagged with a high-confidence level (Fig. S12). Finally, Ukraine (missing country) received the median ratio of Europe (R = 17%). This map of ratio has only been used to partition natural & anthropogenic disturbances to calculate an estimate of the percentage of reduction in harvest for the EU-27 to meet their target by 2030.

**Fig. S14. Annual changes in the ratio between natural disturbances and harvests across Europe.** We first discarded low-confidence pixels (Fig. S12), then for each year, we calculated across Europe the aggregated sum of severities associated with natural disturbances (storms & fire) and divided them by the aggregated sum of severities associated with harvests according to the Landsat-based disturbance map of Seidl et al. (2024). The mean value of the timeseries ($R=17\%$) remarkably matches the value reported at a continental scale in the ground-based study of Patacca et al. (2023), 16%. We did not detect a significant trend in the time-series (p = 0.14 > 0.05), which confirms the conclusion of Patacca et al. (2023) that the ratio remains reasonably constant through time.

**Fig. S15.** **Sensitivity test of the DDCM to different starting assumptions.** The entire DDCM procedure has been run on two different starting assumptions: either (i) Trends in AGC loss estimated at 54 km instead of 18 km (panels **A1**,**A2**,**A3** and blue curve in panel **C**), or (ii) Trends in AGC loss estimated with an additive model ($s\left( y \right)=f_{loss}\left( y \right)+\beta$) instead of the multiplicative model ($s\left( y \right)=\beta f_{loss}(y)$) used in the main study (panels **B1**,**B2**,**B3** and red curve in panel **C**). Results are similar between these three models (panel **C**), however the original study assumptions have the highest goodness of fit (lowest RMSE) with reported UNFCCC data at the country level (panels **A** and **B**). We therefore kept the original approach only.

**Figure S16. Proof of concept for the DDCM Model. A.** Figure extracted from the main study showing the five forest sink components in a business-as-usual scenario in the EU-27 (Fig. 4A). The minimization between AGC simulations and UNFCCC observations has been performed across the whole historical period (2010-2021). **B.** Same figure as panel **A**, except that the minimization between AGC simulations and UNFCCC observations has been performed for the year 2010 only. The model has therefore no knowledge of the decline in the sink from 2011 to 2021, however it manages to capture it (due to the disturbance trends). You can observe that the confidence intervals are minimum in 2010 (year of parametrization) but much larger in 2030 (more uncertainties due to less constraining data).

 **Figure S17. Inconsistencies in biomass change detection using remote sensing.** Calculating AGC changes as the year-on-year difference between two consecutive AGC maps from CCI-ESA is unsuitable, as it produces AGC changes that are inconsistent with UNFCCC reports across the recent historical period (blue triangles versus black dots). For instance, this ‘brute force’ approach produces a net loss of AGC in the Continental region from 2017 to 2021 (except in 2020), contradicting the AGC accumulation reported by the UNFCCC for that period. In contrast, the AGC change simulations from the DDCM closely match UNFCCC data across all biogeographical regions.

**Figure S18. Sensitivity analysis of the spatial variability of input variables.** Using a moving window of 54 km (9 pixels of 18 km), anomalies (from linear trends) were calculated for each input parameter to compute a coefficient of dispersion, defined as $f\left( x \right)=\frac{\mathrm{MAD}\left( x \right)}{\mathrm{median}\left( x \right)}$, where MAD is the median absolute deviation. At the country or biogeographical level, parameters were grouped into three ensembles based on their calculated dispersion values: high variability ($f> 0.4$), moderate variability ($f\in[0.2, 0.4]$), and low variability ($f< 0.2$). These groupings determined the jittering magnitudes applied in the Monte-Carlo framework (Fig. S19). The thresholds 0.2 and 0.4 correspond to the tertiles of the distribution of $f$ across all parameters. The variables $b$ and $c$ correspond to forest growth, the variable $s$ and $\tau$ correspond to the severity and return period of the disturbances. The variable $R$ corresponds to the fraction between natural disturbances and harvests, and finally the variable $f_{c}$ correspond to the net forets area.

**Figure S19. Magnitude of jitter.** Each input variable has been jittered to assess the sensitivity of the DDCM carbon sink projection through a Monte-Carlo framework. The variables $b$ and $c$ correspond to forest growth. ${AGC}_{pot}$has been retrieved with the recovery curves to produce physical projections. The variable $s$ and $\tau$ correspond to the severity and return period of the disturbances. The variable $R$ corresponds to the fraction between natural disturbances and harvests, and finally the variable $f_{c}$ correspond to the net forets area. The slopes and intercepts of the linear trends (either in severity, return period or net forest area) have been jittered by the same scalar value (different for each pixel) to avoid non-physical solutions. The minimum and maximum bounds of the jitter (from $\pm10\%$ to $\pm30$%) have been estimated with the sensitivity analysis produced in Fig. S18.

**Fig. S20. UNFCCC reports and DDCM simulations for Albania, Andorra, and Austria.** Each forest sink component is shown separately, negative values are associated with net sources (C release in the atmosphere). Shaded area shows the range (95% CI) obtained from the 1000 Monte-Carlo simulations (for the total forest sink only for vizualization purposes).

**Fig. S21. UNFCCC reports and DDCM simulations for Belarus, Belgium, and Bosnia and Herzegovina.** Each forest sink component is shown separately, negative values are associated with net sources (C release in the atmosphere). Shaded area shows the range (95% CI) obtained from the 1000 Monte-Carlo simulations (for the total forest sink only for vizualization purposes).

**Fig. S22. UNFCCC reports and DDCM simulations for Bulgaria, Croatia, and Czechia.** Each forest sink component is shown separately, negative values are associated with net sources (C release in the atmosphere). Shaded area shows the range (95% CI) obtained from the 1000 Monte-Carlo simulations (for the total forest sink only for vizualization purposes).

**Fig. S23. UNFCCC reports and DDCM simulations for Denmark, Estonia, and Finland.** Each forest sink component is shown separately, negative values are associated with net sources (C release in the atmosphere). Shaded area shows the range (95% CI) obtained from the 1000 Monte-Carlo simulations (for the total forest sink only for vizualization purposes).

**Fig. S24. UNFCCC reports and DDCM simulations for France, Germany, and Greece.** Each forest sink component is shown separately, negative values are associated with net sources (C release in the atmosphere). Shaded area shows the range (95% CI) obtained from the 1000 Monte-Carlo simulations (for the total forest sink only for vizualization purposes).

**Fig. S25. UNFCCC reports and DDCM simulations for Hungary, Ireland, and Italy.** Each forest sink component is shown separately, negative values are associated with net sources (C release in the atmosphere). Shaded area shows the range (95% CI) obtained from the 1000 Monte-Carlo simulations (for the total forest sink only for vizualization purposes).

**Fig. S26. UNFCCC reports and DDCM simulations for Kosovo, Latvia, and Liechtenstein.** Each forest sink component is shown separately, negative values are associated with net sources (C release in the atmosphere). Shaded area shows the range (95% CI) obtained from the 1000 Monte-Carlo simulations (for the total forest sink only for vizualization purposes).

**Fig. S27. UNFCCC reports and DDCM simulations for Lithuania, Luxembourg, and Moldova.** Each forest sink component is shown separately, negative values are associated with net sources (C release in the atmosphere). Shaded area shows the range (95% CI) obtained from the 1000 Monte-Carlo simulations (for the total forest sink only for vizualization purposes).

**Fig. S28. UNFCCC reports and DDCM simulations for Montenegro, Netherlands, and North Macedonia.** Each forest sink component is shown separately, negative values are associated with net sources (C release in the atmosphere). Shaded area shows the range (95% CI) obtained from the 1000 Monte-Carlo simulations (for the total forest sink only for vizualization purposes).

**Fig. S29. UNFCCC reports and DDCM simulations for Norway, Poland, and Portugal.** Each forest sink component is shown separately, negative values are associated with net sources (C release in the atmosphere). Shaded area shows the range (95% CI) obtained from the 1000 Monte-Carlo simulations (for the total forest sink only for vizualization purposes).

**Fig. S30. UNFCCC reports and DDCM simulations for Romania, Russia (Kaliningrad Oblast province), and Serbia.** Each forest sink component is shown separately, negative values are associated with net sources (C release in the atmosphere). Shaded area shows the range (95% CI) obtained from the 1000 Monte-Carlo simulations (for the total forest sink only for vizualization purposes).

**Fig. S31. UNFCCC reports and DDCM simulations for Slovakia, Slovenia, and Spain.** Each forest sink component is shown separately, negative values are associated with net sources (C release in the atmosphere). Shaded area shows the range (95% CI) obtained from the 1000 Monte-Carlo simulations (for the total forest sink only for vizualization purposes).

**Fig. S32. UNFCCC reports and DDCM simulations for Sweden, Switzerland, and Ukraine.** Each forest sink component is shown separately, negative values are associated with net sources (C release in the atmosphere). Shaded area shows the range (95% CI) obtained from the 1000 Monte-Carlo simulations (for the total forest sink only for vizualization purposes).

**Fig. S33. UNFCCC reports and DDCM simulations for United Kingdom.** Each forest sink component is shown separately, negative values are associated with net sources (C release in the atmosphere). Shaded area shows the range (95% CI) obtained from the 1000 Monte-Carlo simulations (for the total forest sink only for vizualization purposes).
